# The cost effectiveness of personalized dietary advice to increase protein intake in older adults with lower habitual protein intake: a randomized controlled trial

**DOI:** 10.1007/s00394-021-02675-0

**Published:** 2021-10-05

**Authors:** Ilse Reinders, Marjolein Visser, Satu K. Jyväkorpi, Riikka T. Niskanen, Judith E. Bosmans, Ângela Jornada Ben, Ingeborg A. Brouwer, Lothar D. Kuijper, Margreet R. Olthof, Kaisu H. Pitkälä, Rachel Vijlbrief, Merja H. Suominen, Hanneke A. H. Wijnhoven

**Affiliations:** 1 Department of Health Sciences, Faculty of Science, and The Amsterdam Public Health Research Institute, Vrije Universiteit Amsterdam, De Boelelaan 1105, Room O-533, 1081 HV Amsterdam, The Netherlands; 2 Department of General Practice and Primary Health Care, and Helsinki University Central Hospital, Unit of Primary Health Care, University of Helsinki, Helsinki, Finland

**Keywords:** Protein intake, Physical functioning, RCT, Protein recommendation, 400 m walk, Timing

## Abstract

**Purpose:**

To examine the cost effectiveness of dietary advice to increase protein intake on 6-month change in physical functioning among older adults.

**Methods:**

In this multicenter randomized controlled trial, 276 community-dwelling older adults with a habitual protein intake < 1.0 g/kg adjusted body weight (aBW)/d were randomly assigned to either Intervention 1; advice to increase protein intake to ≥ 1.2 g/kg aBW/d (PROT, *n* = 96), Intervention 2; similar advice and in addition advice to consume protein (en)rich(ed) foods within half an hour after usual physical activity (PROT + TIMING, *n* = 89), or continue the habitual diet with no advice (CON, *n* = 91). Primary outcome was 6-month change in 400-m walk time. Secondary outcomes were 6-month change in physical performance, leg extension strength, grip strength, body composition, self-reported mobility limitations and quality of life. We evaluated cost effectiveness from a societal perspective.

**Results:**

Compared to CON, a positive effect on walk time was observed for PROT;  – 12.4 s (95%CI,  – 21.8 to  – 2.9), and for PROT + TIMING;  – 4.9 s (95%CI,  – 14.5 to 4.7). Leg extension strength significantly increased in PROT (+ 32.6 N (95%CI, 10.6–54.5)) and PROT + TIMING (+ 24.3 N (95%CI, 0.2–48.5)) compared to CON. No significant intervention effects were observed for the other secondary outcomes. From a societal perspective, PROT was cost effective compared to CON.

**Conclusion:**

Dietary advice to increase protein intake to ≥ 1.2 g/kg aBW/d improved 400-m walk time and leg strength among older adults with a lower habitual protein intake. From a societal perspective, PROT was considered cost-effective compared to CON. These findings support the need for re-evaluating the protein RDA of 0.8 g/kg BW/d for older adults.

**Trial registration:**

The trial has been registered at ClinicalTrials.gov (NCT03712306). Date of registration: October 2018. Registry name: The (Cost) Effectiveness of Increasing Protein Intake on Physical Functioning in Older Adults. Trial Identifier: NCT03712306.

**Supplementary Information:**

The online version contains supplementary material available at 10.1007/s00394-021-02675-0.

## Introduction

The current EFSA recommended daily allowance (RDA) for protein intake is 0.83 g/kg body weight (BW)/day) (d) for all European adults [1]. However, based on evidence from short term metabolic [2–7] and epidemiological studies [8–15], several expert groups have suggested that the RDA for protein intake should be increased to ≥ 1.0–1.2 g/kg BW/d for older adults to help maintain and regain muscle mass, muscle strength and physical function [16, 17]. Thereby, several national guidelines already increased their RDA, i.e. the RDA of the German-speaking countries (D-A-CH) is increased to 1.0 g/kg BW/d [18], and the Nordic Nutrition Recommendation has increased their RDA to 1.2 g/kg BW/d [19].

The majority of randomized controlled trials (RCTs) on effects of increasing protein intake in older adults showed no benefit on muscle mass, strength or function, but no study specifically targeted those with a lower habitual protein intake [20–22]. Three recent RCTs have investigated the effect of increasing protein intake to ≥ 1.2 g/kg BW/d in healthy older adults with a habitual protein intake below 1.0 g/kg BW/d on lean body mass, strength or physical performance, with inconclusive results [23–25]. Furthermore, previous trials solely investigated the effect of the advice to increase protein intake, and did not take into account the timing of protein intake in close proximity of usual physical activity, which may additionally stimulate muscle protein synthesis (MPS) [26]. Also, no previous trial investigated the cost effectiveness of dietary advice to increase protein intake.

Therefore, the PROMISS trial examined the cost effectiveness of personalized dietary advice aimed to increase protein intake to ≥ 1.2 g/kg adjusted (a) BW/d with or without advice to time protein intake in close proximity of usual physical activity, on change in physical functioning measured by 400-m walking time after 6 months among community-dwelling older adults with a habitual protein intake < 1.0 g/kg aBW/d.

## Methods and subjects

### Summary PROMISS study design

The PROMISS RCT was performed at two study sites; the University of Helsinki, Finland and the Vrije Universiteit Amsterdam, the Netherlands. The first participant was randomized on November 4, 2018 and the last participant completed the study on July 31, 2020. Participants were community-dwelling older adults (≥ 65 y) with a habitual protein intake < 1.0 g/kg aBW/d at baseline, assessed using the Protein Screener 55 + (Pro55 + , www.proteinscreener.nl/#/) [27] followed by a full dietary assessment using food diaries followed by a 24-h dietary recall on three days in those who screened positive (i.e. those with a higher risk on a lower protein intake). We applied adjusted BW depending on participants’ age and BMI. We used adjusted body weight, because underweight persons require extra protein to build muscle tissue, while in overweight persons, much ‘extra weight’ is adipose tissue. For those with a BMI > 25.0–32.0 kg/m^2^ (age ≤ 70 y) or > 27.0–32.0 kg/m^2^ (age > 70 y), we applied aBW corresponding to a BMI of, respectively, 25.0 or 27.0 kg/m^2^. For those with a BMI < 22.0 (age > 70 y), we applied aBW corresponding to a BMI of 22.0 kg/m^2^. For those with a BMI > 18.5–25.0 kg/m^2^ (age ≤ 70 y) or > 22.0–27.0 kg/m^2^ (age > 70 y), we did not adjust BW [28]. For the recommended protein intake, we applied adjusted BW which was based on baseline measured BW.

Detailed information on the trial design, recruitment (the metropolitan area of Finland including Helsinki, Espoo, Vantaa, Kauniainen, and the Netherlands including urban and rural areas) and eligibility criteria can be found in the study protocol [29]. Ethical approval was provided by the Ethics Committee of both sites. Oral informed consent was obtained from participants before the screening procedure and written informed consent was obtained at the start of the first clinic visit.

### Randomization, allocation and masking

**Fig. 1:**
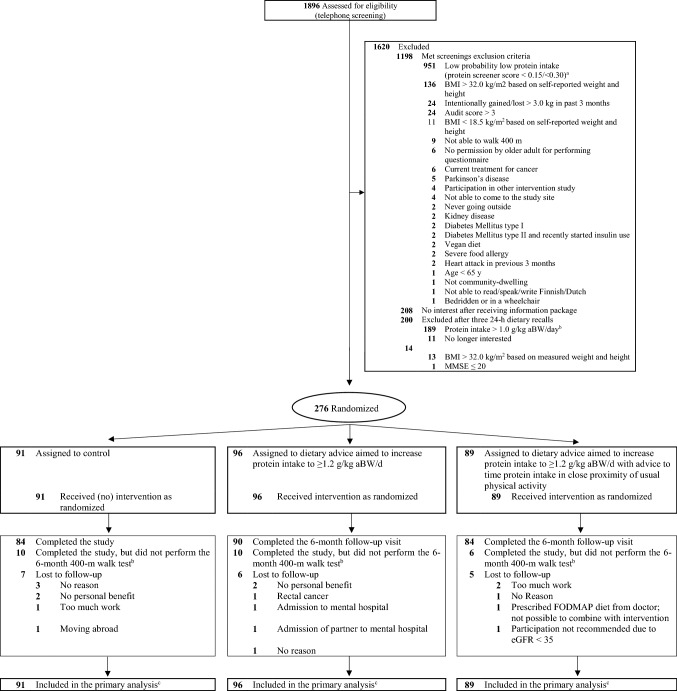
Randomization and participants flow of the PROMISS Randomized Clinical Trial. ^a^Cut-off values of the Pro55+ screener depended on response rates; when response rates to recruitment strategies were low, the cut-off of > 0.15 was applied, while when response rates to recruitment strategies were high, the cut-off of > 0.30 was applied. ^b^Due to the spread of COVID-19 some participants did not want to come to the clinic to perform the measurements, and measured data were therefore missing. Data collected by questionnaires were obtained. ^c^Missing data were imputed via multiple imputation for the primary analysis of change in 400-m walk time

The baseline assessment was performed when all eligibility criteria were met (Fig. 1), after which participants were randomized to one of the three study groups in a 1:1:1 ratio by means of a block randomization procedure at each study site, stratified according to baseline habitual protein intake (< 0.9 or 0.9–1.0 g/kg aBW/d, with aBW based on self-reported BW) and sex. Randomization was performed by an independent statistician, and allocation was done by the nutritionist at the end of the baseline assessment. Due to the nature of the study, researchers, nutritionists and participants were not blinded to the study group. Statistical analyses on all outcomes were carried out by two independent statisticians.

### Intervention

Participants in Intervention 1 (PROT) received a personalized dietary advice face-to-face by nutritionists with the aim to increase protein intake to ≥ 1.2 g/kg aBW/d without increasing energy intake. The dietary advice also included the advice to consume at least one daily meal containing of ≥ 35 g protein, as studies have shown that a greater amount of protein in a meal stimulates protein anabolism in older adults [6, 30, 31]. The advice included the use of regular protein-rich food products that could be purchased by respondents themselves and protein enriched food products freely provided by the research team. The protein enriched food products (including their protein content) were protein bars (23.2 g/100 g; 10.4 g/portion), cereals (16.7 g/100 g; 8.9 g/portion), puddings (10.5 g/100 g; 15.8 g/portion), coconut water (6.1 g/100 g; 20.1 g/portion) and whey powder (87.2 g/100 g; 5.2 g/portion). Participants in Intervention 2 (PROT + TIMING) additionally received personalized advice to consume at least 7.5–10 g protein through protein (en)rich(ed) food products within half an hour after performing usual physical activity. Usual physical activity was defined as either physical exercise (e.g. biking, swimming, tennis) or the most intensive activities of daily living when the participant did not engage in physical exercise (e.g. gardening, housekeeping, doing groceries) for a minimum of 30 min. The advice was linked to the physical activity that was most intense or with the longest duration of the day. Participants were instructed to shift their physical activity or protein intake moment to adhere to this advice, but not to become more or less physically active.

Participants allocated to control (CON) did not receive any advice but were contacted at similar time points as PROT and PROT + TIMING to ask how they were doing.

Follow-up assessments at the clinic took place at 3- and 6-month follow-up. Other contact moments included phone calls that took place in week 2, 4, 8, 16 and 20, where participants from the PROT and PROT + TIMING groups could ask for e.g. clarification of the dietary advice, and participants from CON were asked how they were doing. All participants, by study group, were invited to at least one lecture on non-health-related themes during the trial to stimulate their commitment.

As the last phase of the trial was ongoing during the spread of COVID-19, we deviated from the original study protocol. From March 16 onwards, data collection for the 6-month follow-up visit of the final 80 participants was conducted by means of questionnaire during a phone call at the planned date. The physical measurements were postponed until a maximum of 2 months after the phone call. In the meantime, we asked participants to extend their participation and to continue adhering to the dietary advice or control condition. See ClinicalTrials.gov for a full description of the COVID-19-related amendments.

### Outcomes

The primary outcome was 6-month change in time needed to walk 400 m (Long Distance Corridor Walk) [32, 33]. For all participants, the test begun with a mandatory 40-m walk (warm-up) at their usual pace. For the actual 400-m test (i.e. 10 complete rounds), older adults were instructed to walk as fast as possible at a pace they could maintain for 400 m. Time was recorded to the nearest second. Secondary outcomes included 6-month change in physical performance (Short Physical Performance Battery (SPPB) score, range 0–12, with higher scores indicating better physical functioning [34].), leg extension strength (N), hand-grip strength (Seahan digital hand dynamometer, kg), body composition (body fat percentage (%) and fat-free mass (kg) assessed by bioelectrical impedance analyses (BodyStat 1500MDD, Bodystat Ltd, Douglas, Isle of Men, United Kingdom), and fat percentage and fat-free mass assessed by air displacement plethysmography (BODPOD, COSMED Benelux BV, Nieuwegein, the Netherlands) (BODPOD, COSMED Benelux BV, Nieuwegein, the Netherlands) (Dutch study site only)), self-reported mobility limitations ((3- and 6-month follow-up) two consecutive reports having any difficulty walking 400 m or climbing 10 steps due to a health or a physical problem.), health-related quality of life and healthcare and informal care costs. Health-related quality of life was measured using the EuroQol 5D-5L questionnaire (EQ-5D-5L) [35], and valued using the Dutch value set to calculate utility values (range 0.466 to 1, with higher values indicating better quality of life) [36]. Utility values were used to calculated Quality-adjusted life year (QALY) [37]. The Finnish site used an unofficial translation of the EQ-5D-5L (see Supplement questionnaire). Intervention costs consisted of 3 working-hours of dieticians (1 working-hour = €33) [38] which is comparable to costs incurred in real life/general settings. Protein-rich food products provided were free of costs and, therefore, not included in the intervention costs. Healthcare and informal care use was collected at baseline and at 3- and 6-month follow-up using a modified version of the Resource Utilisation in Dementia Questionnaire [39]. Costs were calculated according to Dutch guidelines [38, 40] and summed into healthcare costs (i.e., costs related to primary and secondary care, and medication use) and societal costs (i.e., healthcare costs and informal care costs) over the period of the study (supplementary eTable7). Lost productivity costs were not included, because all participants were at a pensionable age by design.

### Other measures

Body weight was measured at each clinic visit without shoes in underwear to the nearest 0.1 kg using a digital calibrated scale (Finland; SECA 877, the Netherlands; Marsden M-520). At baseline, body height was measured to the nearest millimeter using a SECA stadiometer for mobile height measurements (Finland; SECA 217, the Netherlands; SECA 214). Corrections were made to adjust the measured body weight for clothing or a cast (minus 1 kg for each element) (baseline; *N* = 3, 3-month follow-up visit, *N* = 15, 6-month follow-up visit, *N* = 28). Appetite was assessed at baseline and the 6-month follow-up visit with three items about appetite and satiety from the Simplified Nutritional Appetite Questionnaire (SNAQ) [41], and one question about the amount of meals and snacks consumed per day. Dietary intake was assessed prior to each clinic visit by means of a full dietary assessment using food diaries on three days, followed by a 24-h dietary recall to assess habitual protein intake. The dietary assessments were used to assess compliance to the dietary advice (≥ 1.2 g protein /kg aBW/d). Among participants of the intervention groups, appreciation and adherence of the intervention and participants’ intention to follow the dietary advice in the future was assessed at the end of the 6-month follow-up visit. Physical activity was objectively measured by means of a 3-axis accelerometer (Axivity AX3®; AXIVITY® Ltd., Newcastle upon Tyne, United Kingdom). Participants were asked to wear the accelerometer on their right thigh for 7 consecutive days after each clinic visit without removing it. The accelerometer was attached by a nutritionist and positioned midway between the anterior superior iliac spine and the patellar tendon and attached using a transparent film (hypoallergenic Tegaderm foam adhesive dressing), allowing participants to perform any activity including swimming and bathing. Accelerometers were initialized to sample at 30 Hz (range ± 8) using Open Movement OmGui Software (version 1.0.0.43). Each device was programmed to record data from 00:00 on the day after the visit to 23:59 seven days later. Minimum wear-time of 22 h per day and at least 4 days (3 weekdays, 1 weekend day) were used as criteria for valid data. The following metrics for the accelerometers were used:

- cpm_24: Average count per minute for 24 h measurement
- total counts_24: Total counts for 24 h measurement
- cpm_pa_24: Average counts per minute of activity above 100 counts for 24 h measurement
- Steps: Total step counts for 24 h measurements
- Time in non-sedentary activities (mins): Time in non-sedentary behavior is the sum of time in move, walking, running and biking category based on the classification defined by Skotte et al. [42]

Please see the study protocol [29] for detailed information of all outcomes (primary, secondary and other), measurements and operationalization.

### Adverse events

In case any (medical) questions raised during the screening or intervention period, participants could consult an independent medical doctor. All adverse events and serious adverse advents were tracked by the nutritionists during the follow-up phone calls, 3-month follow-up visit and 6-month follow-up visit to assess their potential relationship to the intervention at both sites and were documented in the final report. Adverse events were reported within 7 days (death or life threatening situations) or within 15 days (in case of other adverse events) of first knowledge to The Medical Ethical Committee of the Amsterdam UMC, location VUmc (required for the Dutch site only).

### Side effects

We investigated whether the intervention had side effects on appetite, body weight and physical activity.

### Sample size

The study was powered to detect a meaningful change of 28 s (SD = 61 s) [43] between the PROT and PROT + TIMING groups and CON on the primary outcome 400-m walk time, assuming a two-sided test at α = 0.05 with a power of 0.8. For this, 75 participants per study group were needed. Anticipating a drop-out of 15%, the total number of study participants to be included was *n* = 264, or *n* = 44 per study group per site.

### Statistical analyses

The main analyses were based on the intention-to-treat principle. The interventions’ effect on the primary outcome compared with CON was analyzed using a linear regression model. The primary outcome was included in the model as the dependent variable and the study group as the independent one with an adjustment for baseline values of the primary outcome. Multilevel analyses were not necessary, because the intraclass correlation coefficient was small (ICC = 0.00001) [44]. Effect modification was tested by baseline protein intake, sex and baseline 400-m walk time. Residual confounding was checked for baseline 400-m walk time, baseline protein intake, sex and study site (depending on potential effect modification), and was considered present when the regression coefficients of the effect estimate changed more than 10%. Effects, i.e. differences in 6-month changes, were reported as regression coefficients (β) or odd ratios (OR) including 95% confidence intervals (CI) which were 2-sided. Effects were also expressed in Cohen’s d; a result 0.2 or smaller represents a small effect size, 0.5 a medium, and 0.8 or larger a large effect size [45, 46], where CON was the reference group. The interventions’ effect on secondary outcomes and other measures was analyzed analogously to the primary outcome. Planned analyses on incident malnutrition, incident frailty and incident sarcopenia were not performed due to too few incident cases over the follow-up period of 6 months: *N* = 6 (2.3%), *N* = 5 (2.0%), and *N* = 4 (1.5%) for malnutrition, frailty and sarcopenia, respectively.

Multiple Imputation by Chained Equations was used to impute missing data on all outcomes. Variables associated with missingness, outcomes and potential confounders were included in the imputation model. Ten datasets were needed to keep a loss of efficiency below 5%. Imputed estimates were pooled using Rubin’s rules [47].

### Cost-effectiveness analysis (CEA)

CEA was performed from a societal perspective. Differences in total costs and effects (i.e., change in 400-m walk time and QALY) between interventions and CON at 6-month follow-up were analyzed using bivariate regression models [48]. Bias-corrected accelerated bootstrapping with 5.000 replications was used to estimate uncertainty surrounding cost and effect differences. Predictive Mean Matching was used in the imputation procedure to account for the skewed distribution of the costs [49]. The probability of the interventions being cost-effective compared to CON was estimated using a range of willingness-to-pay (WTP) thresholds [50]. For QALYs, a WTP threshold of €20.000/QALY gained was used as recommended by the Dutch Health Care Institute [51].

### Sensitivity analyses

Four sensitivity analyses (SA) were performed to check the robustness of results. SA1 consisted of a per-protocol analyses in which effect estimates were calculated for participants from the PROT and PROT + TIMING groups who reached the protein target of ≥ 1.2 g/kg aBW/d at both 3- and 6-month follow-up vs. all participants from CON. SA2 excluded the 80 participants with an extended 6-month clinic visit due to COVID-19. SA3 included a complete case analysis for each specific outcome (i.e. having both baseline and 6-month follow-up data). SA4 was a CEA from a healthcare perspective. Data were analyzed using SPSS (IBM SPSS Statistics. Armonk, NY) and StataSE 16® (StataCorp LP, CollegeStation, TX, US).

## Results

From October 11, 2018, to October 29, 2019, 1896 older adults underwent telephone screening. A total of 290 people were invited to the clinic to check the final eligibility criteria. Finally, 276 older adults (Finland; *n* = 144, the Netherlands; *n* = 132) who completed the baseline assessment were randomly allocated to one of the three study groups: CON group (*n* = 91), PROT (*n* = 96) and PROT + TIMING (*n* = 89) (Fig. 1). The first participant was randomized on November 4, 2018 and the last participant completed the study on July 31, 2020. Baseline characteristics are presented in Table 1.

**Table 1:** Baseline sample characteristics of the PROMISS trial stratified by study group

	CON *N* = 91	PROT *N* = 96	PROT + TIMING *N* = 89
*Demographics*			
Age, y	75.0 ± 4.4	75.9 ± 5.0	74.6 ± 4.7
Women	50 (54.9)	50 (52.1)	48 (53.9)
BMI, kg/m^2^	26.9 ± 2.9	26.3 ± 2.9	26.7 ± 2.7
MMSE score	28.4 ± 1.7	28.3 ± 1.7	28.5 ± 1.4
Predicted probability score^a^	0.59 ± 0.22	0.59 ± 0.21	0.59 ± 0.21
*Smoking status*			
Never	80 (87.9)	85 (88.5)	82 (92.1)
Former	8 (8.8)	8 (8.3)	5 (5.6)
Current	3 (3.3)	3 (3.1)	2 (2.2)
*Education*^*b*^			
Lower education	5 (5.5)	5 (5.2)	1 (1.1)
Middle education	22 (24.2)	18 (18.8)	15 (16.9)
Higher education	64 (70.3)	73 (76.0)	73 (82.0)
*Household*			
Living alone	36 (39.6)	28 (29.1)	28 (31.5)
I live together with someone	55 (60.4)	68 (70.8)	61 (68.5)
*Self-perceived health*			
(Very) poor	–	–	–
Not poor/not good	18 (19.8)	19 (19.8)	23 (25.8)
(Very) good	73 (80.3)	87 (80.2)	66 (74.2)
*Health in comparison to peers*			
(Much) worse	3 (3.3)	–	3 (3.4)
Not worse/not better	21 (23.1)	25 (26.0)	23 (25.8)
Good	49 (53.8)	58 (60.4)	47 (52.8)
Much better	18 (19.8)	13 (13.5)	16 (18.0)

Data are mean ± SD or N (%)

^a^Predicted probability score (range 0–1) indicates risk of having a protein intake below 1.0 g/kg aBW/d, with higher scores indicating a greater risk on a true lower protein intake

^b^Lower education; elementary education or less, Middle education; lower vocational education and general intermediate, Higher education; intermediate vocational education,

general secondary, higher vocational, college or university

Abbreviations: *BMI* body mass index, *MMSE* mini-mental state examination

CON = no intervention; PROT = personalized dietary advice aimed at increasing protein intake to at least 1.2 g/kg aBW/d; and PROT + TIMING = personalized dietary advice aimed at increasing protein intake to at least 1.2 g/kg aBW/d plus advice to time protein intake in close proximity of usual physical activity

### Attrition

A total of 17 participants (6.2%) dropped-out prior to the 3-month follow-up visit, and one participant prior to the 6-month follow-up visit, which corresponds to a total drop-out rate of 6.5%. There were no significant differences in drop-out rate between study groups. The number of missing observations at month 6 did not differ across study groups for the primary outcome (*P*-value = 0.50) and any of the secondary outcomes. Supplemental Table 1 presents baseline characteristics stratified by participants with complete (*n* = 232) and incomplete (*n* = 44) data on the primary outcome.

### Compliance

**Fig. 2:**
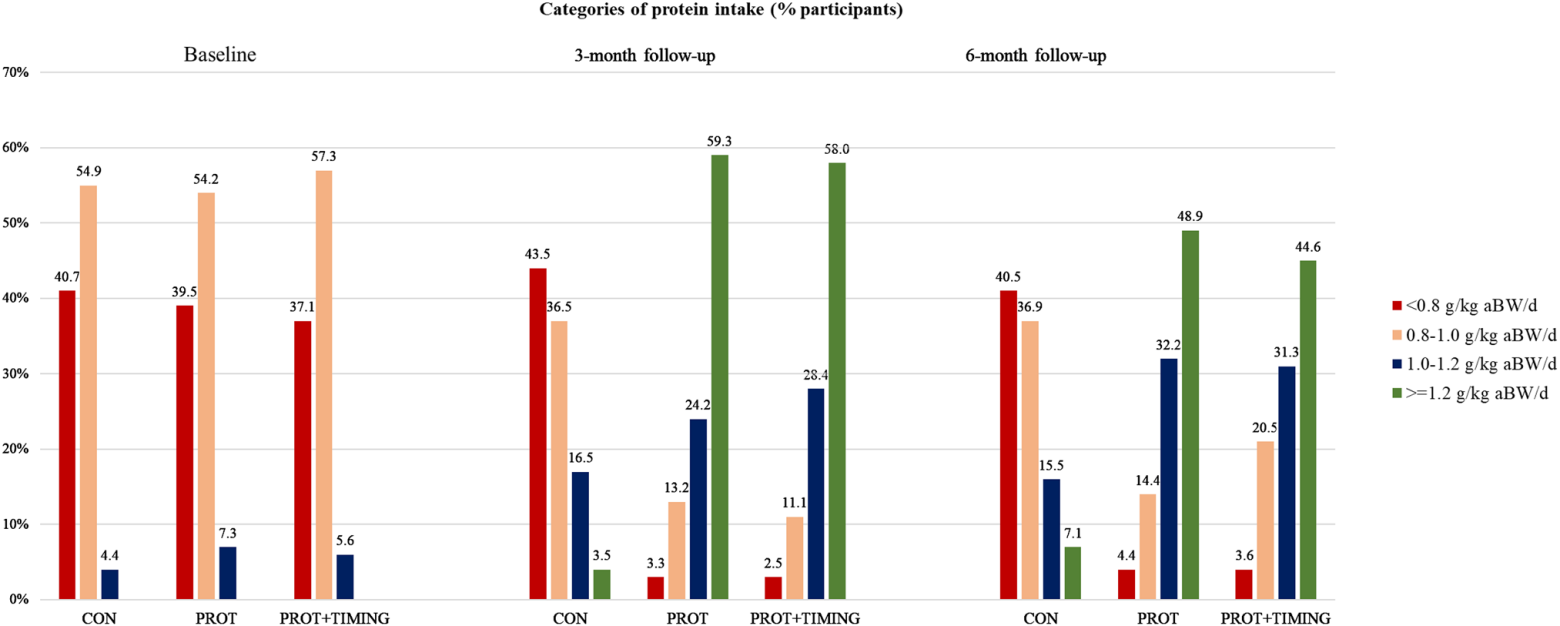
Categories of protein intake at 3 time points during the PROMISS trial. Protein intake was expressed in grams per kilogram adjusted body weight per day (g/kg aBW/d). Participants were included when habitual protein intake was < 1.0 g/kg aBW/d). This was based on self-reported BW during screening. The figure present protein intake based on measured body weight at baseline, 3- and 6-month follow-up. CON = no intervention; PROT = personalized dietary advice aimed at increasing protein intake to at least 1.2 g/kg aBW/d; and PROT+TIMING = personalized dietary advice aimed at increasing protein intake to at least 1.2 g/kg aBW/d and advice to time protein intake in close proximity of usual physical activity

Energy and protein intake at each clinic visit for each study group is presented in Table 2. A statistically significant increase in energy intake was observed for participants from the PROT and PROT + TIMING groups (6-month change; 107 kcal (95% CI, 3–211) and 179 kcal (95% CI, 73–286), respectively), compared to CON. Protein intake increased for PROT (6-month change; 25.5 g (95% CI, 19.9–31.0) and 0.34 g/kg aBW/d (95% CI, 0.27–0.43)) and for PROT + TIMING (6-month change; 25.3 g (95% CI, 19.6–30.9) and 0.34 g/kg aBW/d (95% CI, 0.26–0.41)), compared to CON. Compliance of study participants to adhere to the advice to increase protein intake was indicated by the percentage of participants reaching a certain protein intake (< 0.8 g/kg aBW/d, 0.8–1.0 g/kg aBW/d, 1.0–1.2 g/kg aBW/d or ≥ 1.2 g/kg aBW/d) for each study group at each clinic visit is presented in Fig. 2.

**Table 2:** Protein and energy intake at 3- and 6-month follow-up per study group

	CON *N* = 91	PROT *N* = 96	PROT + TIMING N = 89
*Energy intake, kcal/d*			
Baseline	1574 ± 32	1644 ± 40	1657 ± 40
3-month follow-up	1630 ± 39	1836 ± 42	1913 ± 49
3-month change, β (95% CI)	–	135 (33; 237)	200 (96; 303)
6-month follow-up	1624 ± 38	1802 ± 37	1887 ± 45
6-month change, β (95% CI)	–	107 (3; 211)	179 (73; 286)
*Protein intake, g/d*			
Baseline	60.5 ± 1.2	60.4 ± 1.3	60.4 ± 1.2
3-month follow-up	62.9 ± 1.6	91.0 ± 2.4	92.0 ± 2.1
3-month change, β (95% CI)	–	28.2 (23.0; 33.3)	29.2 (23.8; 34.6)
6-month follow-up	63.7 ± 1.2	89.1 ± 2.3	88.9 ± 2.2
6-month change, β (95% CI)	–	25.5 (19.9; 31.0)	25.3 (19.6; 30.9)
*Protein intake, g/kg aBW/d*			
Baseline	0.82 ± 0.01	0.82 ± 0.01	0.81 ± 0.01
3-month follow-up	0.85 ± 0.02	1.23 ± 0.03	1.23 ± 0.02
3-month change, β (95% CI)	–	0.38 (0.31; 0.44)	0.38 (0.31; 0.45)
6-month follow-up	0.86 ± 0.02	1.21 ± 0.03	1.20 ± 0.03
6-month change, β (95% CI)	–	0.34 (0.27; 0.43)	0.34 (0.26; 0.41)

Data are mean ± standard error. Change scores are the 3- and 6-month follow-up value – the baseline value

Change scores are presented as β (95% CI). *β* regression coefficient. *CI* confidence interval

CON (reference category) = no intervention; *PROT *personalized dietary advice aimed at increasing protein intake to at least 1.2 g/kg aBW/d; and PROT + TIMING = personalized dietary advice aimed at increasing protein intake to at least 1.2 g/kg aBW/d plus advice to time protein intake in close proximity of usual physical activity

### Change in 400-m walk time

Mean 6-month change in 400-m walk time was significantly greater in PROT;  – 12.4 s (95% CI,  – 21.8 to  – 2.9) compared to CON, which indicates a beneficial effect of the intervention on physical functioning. A intervention effect in the same direction was observed in PROT + TIMING, but not statistically significant;  – 4.9 s (95% CI,  – 1.45 to 4.7) (Table 3). Cohen’s d indicated a medium (PROT group) and small (PROT + TIMING group) effect size.

**Table 3:** Primary and secondary outcome measures per study group during the PROMISS trial

	CON *N* = 91	PROT *N* = 96	PROT + TIMING *N* = 89
400-m walk test, s			
Baseline	311.1 ± 9.3	311.3 ± 7.2	292.0 ± 4.6
6-month follow-up	318.2 ± 11.0	306.0 ± 6.85	294.2 ± 4.6
6-month change, β (95% CI)	–	– 12.4 ( – 21.8; – 2.9)	– 4.9 ( – 14.5; 4.7)
Cohen’s d		0.51	0.21
SPPB summary score			
Baseline	9.7 ± 0.17	9.8 ± 0.14	10.1 ± 0.12
6-month follow-up	10.0 ± 0.17	10.0 ± 0.14	10.3 ± 0.14
6-month change, β (95% CI)	–	– 0.04 ( – 0.37; 0.30)	– 0.03 ( – 0.40; 0.35)
Cohen’s d		0.04	0.03
Hand grip, kg			
Baseline	29.2 ± 0.96	30.2 ± 1.04	29.4 ± 1.02
6-month follow-up	27.8 ± 0.93	29.3 ± 1.05	28.6 ± 1.07
6-month change, β (95% CI)	–	0.46 ( – 0.55; 1.48)	0.59 ( – 0.42; 1.60)
Cohen’s d		0.16	0.20
Leg extension strength, N			
Baseline	311.4 ± 12.9	309.4 ± 14.5	302.0 ± 14.7
6-month follow-up	295.5 ± 12.4	326.1 ± 14.2	310.5 ± 14.3
6-month change, β (95% CI)	–	32.6 (10.6; 54.5)	24.3 (0.2; 48.5)
Cohen’s d		0.55	0.40
Body fat percentage (BIA), %			
Baseline	33.4 ± 0.72	32.2 ± 0.76	33.0 ± 0.81
6-month follow-up	33.2 ± 0.76	31.8 ± 0.78	32.7 ± 0.85
6-month change, β (95% CI)	–	– 0.16 ( – 1.37; 1.07)	– 0.03 ( – 1.22; 1.15)
Cohen’s d		0.05	0.01
Fat-free mass (BIA), kg			
Baseline	51.8 ± 0.97	52.0 ± 1.06	52.1 ± 1.06
6-month follow-up	52.1 ± 0.99	52.6 ± 1.15	52.5 ± 1.08
6-month change, β (95% CI)	–	0.29 ( – 0.76; 1.35)	0.15 ( – 0.87; 1.18)
Cohen’s d		0.10	0.05
Fat percentage (BODPOD), %^a^			
Baseline	36.0 ± 1.18	35.5 ± 1.16	35.2 ± 1.48
6-month follow-up	36.4 ± 1.28	35.7 ± 1.13	35.3 ± 1.45
6-month change, β (95% CI)	–	– 0.07 ( – 1.68; 1.53)	– 0.29 ( – 1.96; 1.38)
Cohen’s d		0.03	0.11
Fat-free mass (BODPOD), kg ^a^			
Baseline	50.5 ± 1.37	50.9 ± 1.46	50.3 ± 1.72
6-month follow-up	50.9 ± 1.56	51.0 ± 1.58	51.8 ± 1.85
6-month change, β (95% CI)	–	– 0.22 ( – 2.35; 1.90)	1.16 ( – 1.03; 3.35)
Cohen’s d		0.07	0.35
Self-reported mobility limitation			
Two consecutive reports at baseline and 3 months	20 (21.5)	17 (17.9)	14 (15.9)
Two consecutive reports at 3 months and 6 months	16 (17.2)	16 (16.6)	16 (17.6)
6-month change, β (95% CI)	–	0.25 ( – 1.08; 1.58)	0.54 ( – 0.77; 1.86)
6-month change, OR (95% CI)	–	1.28 (0.34; 4.88)	1.72 (0.46; 6.41)

Data are mean ± standard error or N (%). Change scores are the 6-month follow-up value – the baseline value. Changes scores are presented as β (95% CI)

^a^Fat percentage (BODPOD) and Fat-free mass (BODPOD) were only measured in Dutch participants (*N* = 132)

Abbreviations: *β* regression coefficient adjusted for baseline measures of the outcomes, *BIA* bioelectrical impedance analysis, *CI* confidence interval, *OR* odds ratio, *SPPB* Short Physical Performance Battery

CON (reference category) = no intervention; PROT = personalized dietary advice aimed at increasing protein intake to at least 1.2 g/kg aBW/d; and PROT + TIMING = personalized dietary advice aimed at increasing protein intake to at least 1.2 g/kg aBW/d plus advice to time protein intake in close proximity of usual physical activity

**Fig. 3:**
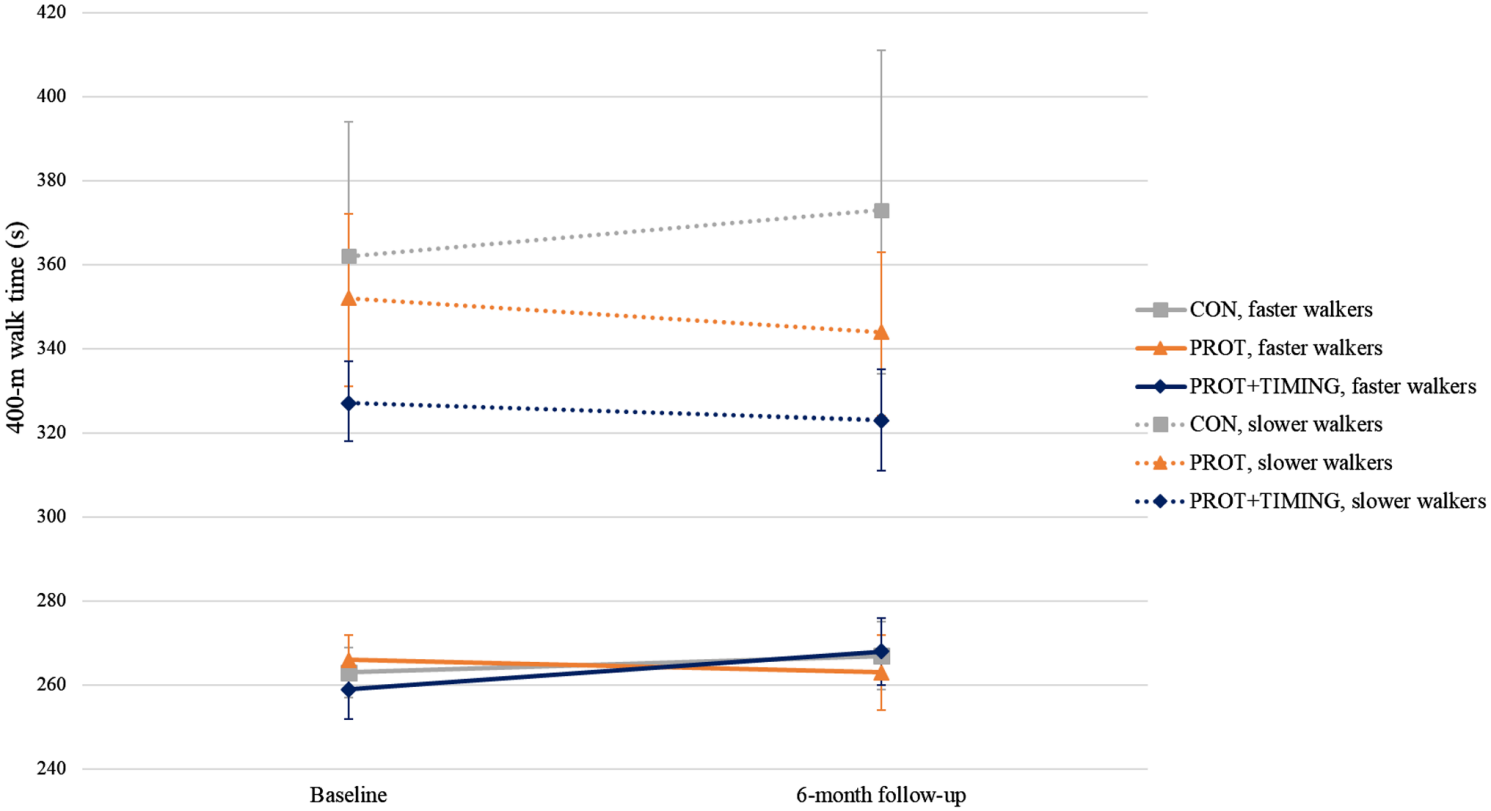
Change in 400-m walk time. Values are means and the bars represent the 95% CI of the mean. CON = no intervention (slower walkers *N* = 44, faster walkers *N* = 47); PROT = personalized dietary advice aimed at increasing protein intake to at least 1.2 g/kg aBW/d (slower walkers *N* = 51, faster walkers *N* = 45); and PROT+TIMING = personalized dietary advice aimed at increasing protein intake to at least 1.2 g/kg aBW/d plus advice to time protein intake in close proximity of usual physical activity (slower walkers *N* = 44, faster walkers *N* = 46)

Baseline 400-m walk time was a statistically significant effect modifier of the PROT + TIMING effect on change in 400-m walk time (*P*-value = 0.0002). Therefore, the main analyses were repeated stratified by median baseline 400-m walk time (294.5 s) and results presented in Fig. 3 and Supplemental Table 2. Among slower walkers, PROT ( – 18.2 s (95% CI,  – 35.4 to  – 1.2)) and PROT + TIMING ( – 15.0 s (95% CI,  – 32.3 to 2.3)) improved in walk time compared to CON, although the latter was not statistically significant. There were no significant intervention effects among faster walkers. No effect modification was observed for baseline protein intake and sex. Baseline protein intake, sex or study site did not confound the intervention effect on change in 400-m walk time and were, therefore, not included in the models.

### Secondary outcomes

Both PROT and PROT + TIMING improved leg extension strength (PROT; 32.6 N (95% CI, 10.6–54.5) and PROT + TIMING; 24.3 N (95% CI, 0.2–48.5)), compared to CON. No intervention effects on other secondary outcomes were found (Table 3). There was no effect modification by baseline 400-m walk time, protein intake or sex for any of the secondary outcomes.

### Cost-effectiveness

**Fig. 4:**
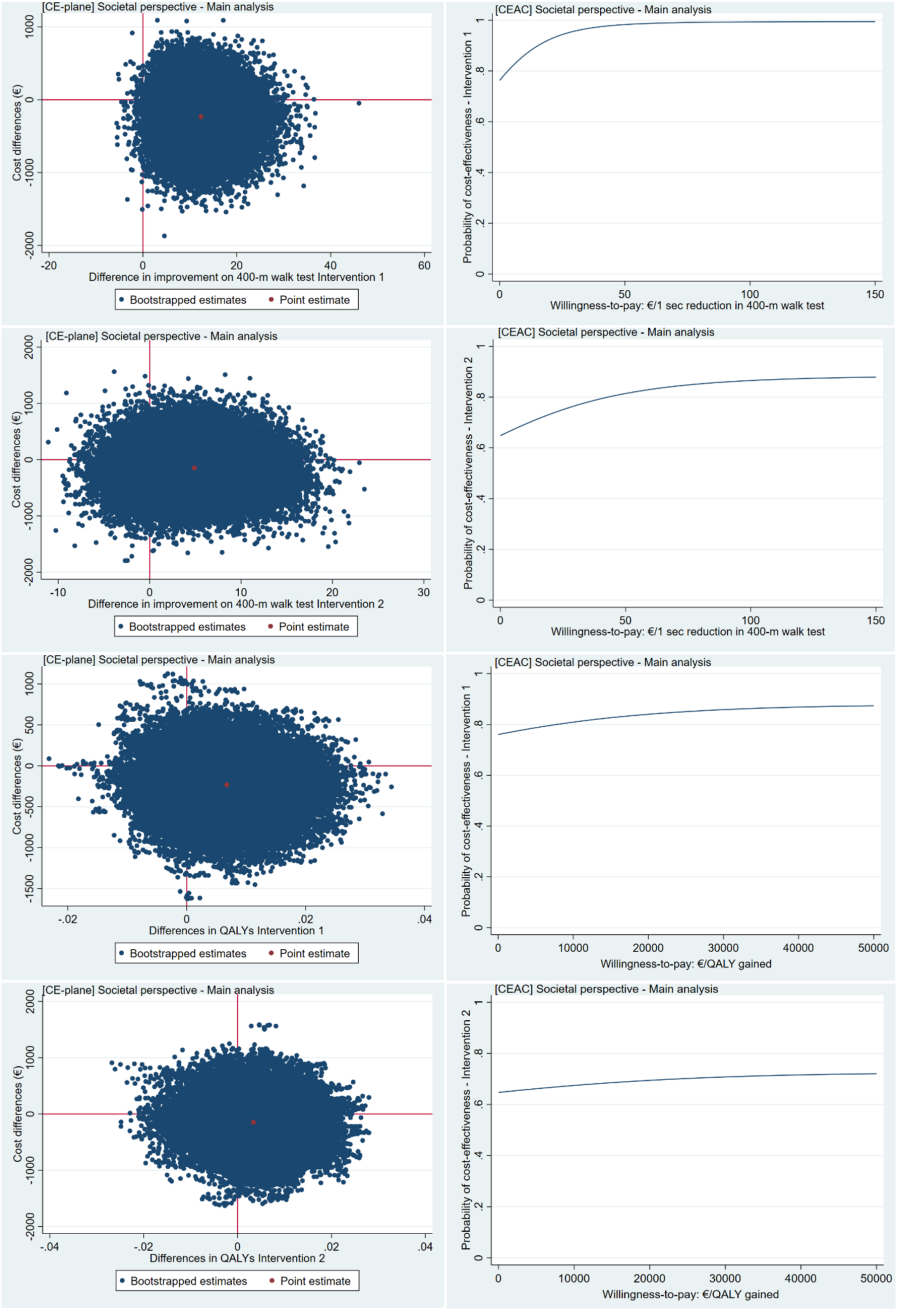
Cost-effectiveness planes (CE-plane) from the societal perspective. Cost-effectiveness planes (CE-plane) from the societal perspective showing the incremental cost-effectiveness ratio point estimate (red dot) and the distribution of the 5000 replications of the bootstrapped cost-effective pairs (blue dots). Cost-effectiveness acceptability curves (CEAC) indicating the probability of cost-effectiveness (y-axis) for different willingness-to-pay (WTP) thresholds per unit of effect gained (x-axis)

Mean effects and costs by study group are presented in Table 4. There were no statistically significant cost differences between both interventions and CON from a societal perspective (PROT; -€227 (95% CI,  – 919 to 357), PROT + TIMING; -€144 (95% CI,  – 861 to 655), Table 5. PROT was significantly more effective on change in 400-m walk time compared to CON (PROT;  – 13 (95% CI,  – 22 to  – 2) while PROT + TIMING was not;  – 7 (95% CI,  – 13 to 3). Most of bootstrapped cost-effective pairs were in southeast quadrant where the interventions were less costly and more effective compared to CON. Similar results were found for QALY outcome. From a societal perspective, probabilities of PROT and PROT + TIMING being cost effective compared to CON increased to 0.84 and 0.69, respectively, at a WTP of 20.000 €/QALY gained (Supplemental Table 3 and Fig. 4).

**Table 4:** Mean effects and costs by study group and mean difference at 6 month follow-up

	CON *N* = 91	PROT *N* = 96	Mean differences 95% CI	PROT + TIMING *N* = 89	Mean differences 95% CI
*Effect*					
400-m walk test, s	318 (10)	306 (6)	– 12 ( – 21; – 2)	294 (4)	– 5 ( – 14; 5)
QALY	0.896 (0.010)	0.910 (0.009)	0.013 ( – 0.013; 0.040)	0.903 (0.010)	0.007 ( – 0.021; 0.034)
*Costs, *€					
Intervention costs	0	99	99 (NA)	99	99 (NA)
Primary care costs	234 ± 43	176 ± 34	– 58 ( – 172; 43)	150 ± 24	– 84 ( – 201; – 6)
Secondary care costs	507 ± 131	487 ± 191	– 20 ( – 367; 612)	661 ± 289	154 ( – 312; 1025)
Medication costs	255 ± 148	108 ± 19	– 147 ( – 737; 18)	76 ± 11	– 179 ( – 795; -19)
Total healthcare costs	998 ± 236	871 ± 196	– 127 ( – 782; 448)	988 ± 289	– 10 ( – 707; 767)
Informal care costs	162 ± 92	56 ± 20	– 106 ( – 438; – 18)	27 ± 10	– 135 ( – 473; – 12)
Total societal costs	1161 ± 256	928 ± 202	– 234 ( – 908; 377)	1015 ± 289	– 146 ( – 850; 646)

Data are mean ± standard error (SE). €, Euros *CI* confidence interval, *QALY* quality-adjusted life-years

Intervention costs consisted of 3 working-hours of nutritionist (1 working-hour = €33)

*CON *no intervention, *PROT *personalized dietary advice aimed at increasing protein intake to at least 1.2 g/kg aBW/d; and PROT + TIMING = personalized dietary advice aimed at increasing protein intake to at least 1.2 g/kg aBW/d plus advice to time protein intake in close proximity of usual physical activity

**Table 5:** Results of the cost-effectiveness analysis from the societal perspective

Effect outcome*	Cost difference, € (95% CI)	Effect difference^§^ * – 1 (95% CI)	ICER €/ effect gained	Distribution of the cost-effectiveness plane			
				North-East	South-East	South-West	North-West
Societal perspective							
Main analysis							
PROT compared to CON							
Improvement in 400-m walk test, s	– 227 ( – 919; 357)	13 (2; 22)	– 17	24%	76%	0%	0%
QALY	– 227 ( – 919; 357)	0.006 (-0.007; 0.020)	– 35,185	18%	66%	11%	5%
PROT + TIMING compared to CON							
Improvement in 400-m walk test, s	– 144 ( – 861; 655)	7 (-3; 13)	– 20	30%	57%	8%	4%
QALY	– 144 ( – 861; 655)	0.005 ( – 0.010; 0.017)	– 14,189	22%	47%	20%	11%
SA1—Per protocol analysis							
PROT compared to CON							
Improvement in 400-m walk test, s	– 361 ( – 1033; 293)	9 ( – 21; 1)	– 39	14%	82%	3%	1%
QALY	– 361 ( – 1033; 293)	0.009 ( – 0.005; 0.28)	– 38,106	12%	79%	6%	3%
PROT + TIMING compared to CON							
Improvement in 400-m walk test, s	– 752 ( – 1499; – 359)	5 ( – 15; 6)	– 148	0	78%	22%	0
QALY	– 752 ( – 1499; – 359)	0.006 ( – 0.014; 0.027)	– 123,520	0%	76%	24%	0%
SA2 – Excluding participants with an extended 6-month follow-up visit due to COVID-19							
PROT compared to CON							
Improvement in 400-m walk test, s	– 332 ( – 1384; 163)	20 (4; 31)	– 17	17%	83%	0%	0%
QALY	– 332 ( – 1384; 163)	0.010 ( – 0.006; 0.026)	– 33,888	14%	75%	8%	3%
PROT + TIMING compared to CON							
Improvement in 400-m walk test, s	– 78 ( – 993; 895)	11 ( – 2; 18)	– 7	41%	55%	3%	1%
QALY	– 78 ( – 993; 895)	0.010 ( – 0.010; 0.025)	− 7921	34%	47%	11%	8%
SA3—Complete case analysis							
PROT compared to CON							
Improvement in 400-m walk test, s	– 174 ( – 648; 249)	11 (3; 19)	– 16	21%	79%	0%	0%
QALY	– 264 ( – 1020; 388)	– 0.007 ( – 0.007; 0.021)	40,077	5%	13%	65%	17%
PROT + TIMING compared to CON							
Improvement in 400-m walk test, s	– 39 ( – 608; 954)	6 ( – 1; 14)	– 6	41%	53%	3%	3%
QALY	– 175 ( – 960; 677)	– 0.005 ( − 0.007; 0.019)	34,409	8%	14%	53%	25%

Data are mean (95% CI). The effect outcome 4000 m walk test was multiplied by  – 1 to keep the cost-effectiveness plane interpretable. *€* Euros *CI* confidence interval, *QALY* quality-adjusted life-years, *ICER* incremental cost-effectiveness ratio

CON (reference category) = no intervention; PROT 1 = personalized dietary advice aimed at increasing protein intake to at least 1.2 g/kg aBW/d; and PROT + TIMING = personalized dietary advice aimed at increasing protein intake to at least 1.2 g/kg aBW/d plus advice to time protein intake in close proximity of usual physical activity

SA1: sensitivity analysis 1, per-protocol analysis including participants from the two intervention groups who reached the protein target of at least 1.2 g/kg aBW/d at both 3- and 6-month follow-up vs. participants from CON (total = 154; CON *N* = 91; PROT *N* = 36, PROT + TIMING *N* = 27).

SA2: sensitivity analysis 2 excluding participants with an extended month-6 clinic visit due to COVID-19 (total = 196, CON *N* = 65, PROT *N* = 64, PROT + TIMING *N* = 67)

SA3: sensitivity analysis 3 using complete cases for 400-m walk test and total societal costs (total = 227, CON *N* = 74, PROT *N* = 77, PROT + TIMING *N* = 76); and using complete cases for QALYs and total societal costs (total = 253, CON N = 84, PROT *N* = 87, PROT + TIMING *N* = 82)

### Sensitivity analyses

The per-protocol analysis (SA1) included 36 participants from PROT (37.5%), and 27 participants from PROT + TIMING (30.3%). Results direction was consistent with the main analyses. However, PROT effect on change in 400-m walk time became statistically non-significant (Supplemental Table 4). SA2 (i.e. excluding the 80 participants with an extended month-6 clinic visit due to COVID-19) and SA3 (i.e. using complete data only) showed similar results as the main analyses (Supplemental Tables 5 and 6, respectively). In SA4 (CEA from a healthcare perspective), the probabilities of PROT and PROT + TIMING being cost effective compared to CON were 0.77 and 0.58, respectively, at a WTP of €20.000/QALY gained (Supplemental Table 7 and Supplemental Fig. 1).

### Adverse events and side effects

A total of twelve adverse events were reported during the intervention period, of which all were judged as unrelated to the study.

Body weight and appetite were not affected by the intervention (Supplemental Table 8). Physical activity did also not change significantly between groups (Supplemental Table 9).

## Discussion

This multicenter RCT included 276 community-dwelling older adults from Finland and the Netherlands with habitual protein intake < 1.0 g/kg aBW/d. Personalized dietary advice resulted in 37.5% of participants from PROT and 30.3% of participants from PROT + TIMING achieving the protein target of 1.2 g/kg aBW/d at the 3- and 6-month follow-up visit. The interventions led to a faster walk time—especially for slower walkers at baseline—and an increase in leg extension strength after 6 months compared to CON, but had no effect on other secondary outcomes. Adding advice to consume protein (en)rich(ed) foods within half an hour after usual physical activity did not result in a stronger nor relevant different intervention effect. From a societal perspective, PROT was considered cost-effective compared to CON.

Six months of dietary advice aiming to increase protein intake to 1.2 g/kg aBW/d among community-dwelling older adults with a habitual protein intake < 1.0 g/kg aBW/d induced better physical functioning. This result was supported by significant increase in leg extension strength. Participants from PROT needed on average 12 s less to complete the 400-m walk test after 6 months compared to CON, and those from PROT and PROT + TIMING with a slower baseline walking speed needed on average 15–18 s less to complete the test, which is considered a small to substantial change [43]. Based on observational data [52], our mean change of 12 s would (PROT) imply a 6% lower mortality and 10% lower mobility disability risk, and a mean change of 17 s among the slower walkers would imply a 8% and 15% lower risk, respectively.

Three previous trials have investigated the effect of dietary advice to increase protein intake on e.g. physical functioning in older adults with a lower habitual protein intake [23–25]. Bhasin et al. observed a positive effect increasing protein intake to 1.3 g/kg/d by means of supplements on fat mass, however, no effect on 6-min walking distance, muscle strength or appendicular and trunk lean mass among functionally limited older men with a mean BMI of 30.3 kg/m^2^ [23]. The lack of effect in this study compared to ours could be explained by the smaller sample size, higher BMI (mean BMI was 30.3 kg/m^2^ compared to a mean BMI of 26.6 kg/m^2^ in our study), and use of unadjusted BW for calculating the protein target, which may have led to an overestimation of their protein requirement as in overweight persons much ‘extra weight’ is adipose tissue and not muscle. Park et al. showed that increasing protein intake to 1.5 g/kg BW/d by means of supplements—but not to 1.2 g/kg BW/d—improved physical performance assessed by 4-m gait speed compared to a protein intake of 0.8 g/kg BW/d in prefrail or frail malnourished older adults with a mean BMI of 24.1 kg/m^2^ [24]. They also reported a positive effect on appendicular skeletal muscle mass, but not on hand-grip strength. In post hoc analyses, we did not observe an effect on 4-m gait speed, neither in the whole sample nor in slower walkers only (data not shown). Our total study population had a higher mean baseline 4-m gait speed (total sample 1.29 m/s, slow walkers 1.18 m/s) compared to 1.0 m/s from Park et al. [24], which is potentially not susceptible for improvement by increasing protein intake. The third RCT of Ten Haaf et al. observed positive effects of 12 weeks of 31 g of protein supplementation on lean body mass (%) and a decrease in fat mass, but no effect on total SPPB score, leg extension or grip strength [25]. These findings may, however, be explained by the high level of aerobic exercise participants were engaged in [53, 54]. In addition, they included physically active older adults with a median baseline SPPB score of 12 (IQR: 11–12). The absence of an intervention effect on physical functioning may have been caused by a ceiling effect of the SPPB. Our non-significant—but still positive—intervention effect in the PROT + TIMING group is possibly due to the fact that the PROT + TIMING group is faster at baseline by chance, as subgroup analyses showed a smaller intervention effect among the participants with a faster baseline walking speed.

One strength of this study is the robustness of the trial design, including block randomization and stratification factors, and the large sample size. We included multiple—for the general older population validated—measures of physical functioning, muscle strength and body composition, as well as participant-reported outcomes such as self-reported health or quality of life. As expert groups recommend protein intake of ≥ 1.0–1.2 g /kg BW/d for all older adults, we included a general older population with a habitual protein intake < 1.0 g/kg aBW/d, enabling generalizability of our study finding to this population. A strength compared to (most) previous studies is that we included older adults with a habitual protein intake < 1.0 g/kg aBW/d and used adjusted BW. Novel aspects were that we examined the simultaneous effect of the timing of protein intake in close proximity of usual physical activity on change in physical functioning and the cost effectiveness of both interventions.

This study has some limitations. First, blinding was not possible because of the nature of the study. Second, we did not assess compliance to the advice regarding the timing of protein intake in close proximity of usual physical activity (PROT + TIMING). Third, participants from both interventions reported a statistical significant increase in energy intake, which was not the intention of the advice, however, no increase in body weight was observed. Fourth, participants were included when protein intake was < 1.0 g/kg aBW/d, which was based on self-reported BW during screening. Based on measured BW at baseline, some participants (5.8%) had a protein intake of > 1.0–1.2 g/kg aBW/d. Fifth, our QoL questionnaire does not specifically focus on physical functioning. Sixth, as with most clinical trials among volunteers, the study population most likely consisted of highly motivated older adults and the intervention may not be effective in a less motivated population. Finally, we acknowledge that the results of secondary outcomes should be considered exploratory as multiple comparisons increase type I error. However, the positive intervention effect on leg extension strength supports the main effect on the primary outcome.

## Conclusions

This RCT highlights the importance of providing dietary advice to increase protein intake to ≥ 1.2 g/ kg aBW/d among community-dwelling older adults with a habitual protein intake < 1.0 g/kg aBW/d, as it was shown to be effective in improving physical function and leg extension strength. Our findings support the need for re-evaluating the protein RDA of 0.8 g/kg BW/d for older adults, but need replication in other studies.

## Supplementary Information

Below is the link to the electronic supplementary material.

Supplementary file1 (DOCX 457 KB)

Supplementary file2 (PDF 174 KB)

## Data Availability

Data described in the manuscript will be made available upon request pending approval of the PROMISS project office.

## Code availability

Code book and analytic code will be made available upon request pending approval of the PROMISS project office.
