# Correction to: The cost effectiveness of personalized dietary advice to increase protein intake in older adults with lower habitual protein intake: a randomized controlled trial

**DOI:** 10.1007/s00394-021-02778-8

**Published:** 2021-12-14

**Authors:** Ilse Reinders, Marjolein Visser, Satu K. Jyväkorpi, Riikka T. Niskanen, Judith E. Bosmans, Ângela Jornada Ben, Ingeborg A. Brouwer, Lothar D. Kuijper, Margreet R. Olthof, Kaisu H. Pitkälä, Rachel Vijlbrief, Merja H. Suominen, Hanneke A. H. Wijnhoven

**Affiliations:** 1 Department of Health Sciences, Faculty of Science, and The Amsterdam Public Health Research Institute, Vrije Universiteit Amsterdam, De Boelelaan 1105, Room O-533, 1081 HV Amsterdam, The Netherlands; 2 Department of General Practice and Primary Health Care, and Helsinki University Central Hospital, Unit of Primary Health Care, University of Helsinki, Helsinki, Finland

## Correction to: European Journal of Nutrition 10.1007/s00394-021-02675-0

In section “Change in 400‑m walk time”, second sentence should read as

A intervention effect in the same direction was observed in PROT + TIMING, but not statistically significant; – 4.9 s (95% CI, – 14.5 to 4.7) (Table 3).

