# Supplementary material for: The cost effectiveness of personalized dietary advice to increase protein intake in older adults with lower habitual protein intake: a randomized controlled trial
Source: Eur J Nutr. 2021 Oct 5;61(1):505–20. doi: 10.1007/s00394-021-02675-0 (PMC8490609; doi:10.1007/s00394-021-02675-0)
Supplement: Supplementary file 1 — Supplementary file1 (DOCX 457 KB) [file 394_2021_2675_MOESM1_ESM.docx]

**The cost-effectiveness of personalized dietary advice to increase protein intake on change in physical functioning in older adults with lower habitual protein intake**

A Randomized Controlled Trial

**European Journal of Nutrition**

Ilse Reinders^1^, Marjolein Visser^1^, Satu K. Jyväkorpi^2^, Riikka T. Niskanen^2^, Judith E. Bosmans^1^, Ângela Jornada Ben^1^, Ingeborg A. Brouwer^1^_,_ Lothar D. Kuijper^1^, Margreet R. Olthof^1^, Kaisu H. Pitkälä^2^, Rachel Vijlbrief^1^, Merja H. Suominen^2^ and Hanneke A.H. Wijnhoven^1^_._

^1^Department of Health Sciences, Faculty of Science, and the Amsterdam Public Health research institute, Vrije Universiteit Amsterdam, The Netherlands; ^2^ University of Helsinki, Department of General Practice and Primary Health Care, and Helsinki University Central Hospital, Unit of Primary Health Care, Finland.

**Corresponding author:**

Hanneke Wijnhoven

E-mail: hanneke.wijnhoven@vu.nl

**Supplementary Table 1.** Baseline characteristics stratified by participants with complete and incomplete data on the primary outcome at 6-month follow-up.

|  | **Complete**  **N=232** | **Incomplete**  **N=44** | **Difference**  **(95% CI)** |
| --- | --- | --- | --- |
| **Demographics** |  |  |  |
| Age, y | 75 ± 4.6 | 76 ± 5.5 | -1.20 (-2.74; 0.33) |
| Women | 116 (50.0) | 32 (72.7) | -22.7 (-37.3; -8)^c^ |
| BMI, kg/m^2^ | 26.5 ± 2.9 | 27.1 ± 1.5 | -0.5 (-1.3; 0.4) |
| MMSE score | 28.4 ± 1.6 | 28.6 ± 1.7 | -0.2 (-0.8; 0.3) |
| Predicted probability score^a^ | 0.60 ± 0.21 | 0.55 ± 0.23 | 0.05 (-0.01; 0.12) |
| Smoking status |  |  |  |
| Never | 108 (46.5) | 38 (63.6) | -17.1 (-32.7; -1.5)^c^ |
| Former | 116 (50.0) | 16 (36.4) | 13.6 (-1.96; 29.2) |
| Current | 8 (3.4) | - | 3.4 (1.1; 5.8)^c^ |
| Education^b^ |  |  |  |
| Lower education | 11 (4.7) | - | 5.0 (0.98; 7.4)^c^ |
| Middle education | 49 (21.1) | 6 (13.6) | 7.5 (-3.9; 18.9) |
| Higher education | 172 (74.1) | 38 (86.4) | -12.3 (-23.9; -0.7)^c^ |
| Household |  |  |  |
| Living alone | 72 (31.0) | 20 (45.4) | -14.4 (-30.3; 0.15) |
| I live together with someone | 160 (69.0) | 24 (54.5) | 14.5 (-1.4; 30.4) |
| Self-perceived health |  |  |  |
| (Very) poor | 148 (63.8) | 28 (63.6) | 0.2 (-15.3; 15.7) |
| Not poor/not good | 47 (20.3) | 13 (29.5) | -9.2 (-23.6; 5.2) |
| (Very) good | 37 (15.9) | 3 (6.82) | 9.1 (0.2; 17.9)^c^ |
| Health in comparison to peers |  |  |  |
| (Much) worse | 6 (2.6) | 0 | 2.6 (0.55; 4.6)^c^ |
| Not worse/not better | 59 (25.4) | 10 (22.7) | 2.7 (-10.9; 16.3) |
| Good | 125 (53.9) | 29 (65.9) | -12 (-27.4; 3.4) |
| Much better | 42 (18.1) | 5 (22.4) | -4.3 (-17.5; 9.0) |

Data are mean ± SD or N (%)

^a^Predicted probability score (range 0-1) indicates risk of having a protein intake below 1.0 g/kg aBW/d, with higher scores indicating a greater risk on a true lower protein intake.

^b^Lower education; elementary education or less, Middle education; lower vocational education and general intermediate, Higher education; intermediate vocational education,

general secondary, higher vocational, college or university.

^c^Statistically significant difference T-test and Chi-squared test.

Abbreviations: BMI; body mass index, CI; confidence interval, MMSE; mini-mental state examination.

**Supplementary Table 2.** Change in primary outcome measure stratified by baseline median 400-m walk time per study group.

|  | **CON** | **PROT** | **PROT+TIMING** |
| --- | --- | --- | --- |
| **400-m walk test, s** |  |  |  |
| **< baseline median 400-m walk time (fast walkers)** | N=47 | N=43 | N=45 |
| Baseline | 263.3 ± 3.1 | 265.7 ± 3.2 | 259.0 ± 3.2 |
| 6-month follow-up | 267.3 ± 4.0 | 263.3 ± 4.4 | 267.6 ± 4.7 |
| 6-month change, β (95% CI) | - | -6.4 (-14.8; 2.0) | 4.6 (-3.7; 12.9) |
| **> baseline median 400-m walk time (slow walkers)** | N=44 | N=53 | N=44 |
| Baseline | 362.2 ± 15.7 | 351.5 ± 10.3 | 327.4 ± 4.8 |
| 6-month follow-up | 372.6 ± 19.2 | 343.7 ± 9.5 | 322.8 ± 6.0 |
| 6-month change, β (95% CI) | - | -18.2 (-35.4; -1.2) | -15.0 (-32.3; 2.3) |

Data are mean ± standard error. Change scores are the 6-month follow-up value – the baseline value. Changes scores are presented as β (95% CI).

Abbreviations: β; regression coefficient adjusted for baseline measures of the outcomes. CI; confidence interval.

CON (reference category) = no intervention; PROT = personalized dietary advice aimed at increasing protein intake to at least 1.2 g/kg aBW/d; and PROT+TIMING = personalized dietary advice aimed at increasing protein intake to at least 1.2 g/kg aBW/d plus advice to time protein intake in close proximity of usual physical activity.

**Supplementary Table 3.** Probability of interventions being cost-effective compared to control group according to a range of willingness-to-pay thresholds.

| **QALY** | **€0/unit of improvement** | | **€20,000/unit of improvement** | | **€50,000/ unit of improvement** | |
| --- | --- | --- | --- | --- | --- | --- |
| **Healthcare perspective** | | | | | | |
| **Main analysis** | | | | | | |
| 400-m walk test, s | | | | | | |
| PROT | 0.66 | | 0.99 | | 0.99 | |
| PROT+TIMING | 0.51 | | 0.87 | | 0.87 | |
| QALY | | | | | | |
| PROT | 0.66 | | 0.77 | | 0.83 | |
| PROT+TIMING | 0.51 | | 0.58 | | 0.63 | |
| **SA1 – Per protocol analysis** | | | | | | |
| 400-m walk test, s | | | | | | |
| PROT | 0.66 | | 0.99 | | 0.99 | |
| PROT+TIMING | 0.51 | | 0.87 | | 0.87 | |
| QALY | | | | | | |
| PROT | 0.73 | | 0.85 | | 0.90 | |
| PROT+TIMING | 0.99 | | 0.98 | | 0.93 | |
| **SA2 – Excluding participants with an extended 6-month follow-up visit due to COVID-19.** | | | | | | |
| 400-m walk test, s | | | | | | |
| PROT | 0.82 | | 0.99 | | 0.99 | |
| PROT+TIMING | 0.49 | | 0.95 | | 0.95 | |
| QALY | | | | | | |
| PROT | | 0.82 | | 0.90 | | 0.93 |
| PROT+TIMING | | 0.49 | | 0.61 | | 0.72 |

| **SA3 – Complete case analysis** | | | | | | | |
| --- | --- | --- | --- | --- | --- | --- | --- |
| 400-m walk test, s | | | | | | | |
| PROT | | 0.60 | | 0.99 | | 0.99 | |
| PROT+TIMING | | 0.99 | | 0.99 | | 0.99 | |
| QALY | | | | | | | |
| PROT | | 0.68 | | 0.52 | | 0.36 | |
| PROT+TIMING | | 0.36 | | 0.36 | | 0.36 | |
| **Societal perspective** | | | | | | |  |
| **Main analysis** | | | | | | |  |
| 400-m walk test, s | | | | | | |  |
| PROT | 0.76 | | 0.99 | | 0.99 | |  |
| PROT+TIMING | 0.65 | | 0.87 | | 0.87 | |  |
| QALY | | | | | | |  |
| PROT | 0.76 | | 0.84 | | 0.87 | |  |
| PROT+TIMING | 0.65 | | 0.69 | | 0.72 | |  |
| **SA1 – Per protocol analysis** | | | | | | |  |
| 400-m walk test, s | | | | | | |  |
| PROT | 0.85 | | 0.96 | | 0.96 | |  |
| PROT+TIMING | 0.99 | | 0.77 | | 0.77 | |  |
| QALY | | | | | | |  |
| PROT | 0.84 | | 0.91 | | 0.93 | |  |
| PROT+TIMING | 0.99 | | 0.99 | | 0.96 | |  |
| **SA2 – Excluding participants with an extended 6-month follow-up visit due to COVID-19.** | | | | | | |  |
| 400-m walk test, s | | | | | | |  |
| PROT | 0.83 | | 0.99 | | 0.99 | |  |
| PROT+TIMING | 0.57 | | 0.95 | | 0.95 | |  |
| QALY | | | | | | |  |
| PROT | 0.83 | | 0.91 | | 0.93 | |  |
| PROT+TIMING | 0.57 | | 0.68 | | 0.76 | |  |

| **SA3 – Complete case analysis** | | | |
| --- | --- | --- | --- |
| 400-m walk test, s | | | |
| PROT | 0.79 | 0.99 | 0.99 |
| PROT+TIMING | 0.99 | 0.30 | 0.99 |
| QALY | | | |
| PROT | 0.78 | 0.65 | 0.45 |
| PROT+TIMING | 0.45 | 0.45 | 0.45 |

PROT = personalized dietary advice aimed at increasing protein intake to at least 1.2 g/kg aBW/d; and PROT+TIMING = personalized dietary advice aimed at increasing protein intake to at least 1.2 g/kg aBW/d plus advice to time protein intake in close proximity of usual physical activity.

**Supplementary Table 4.** Sensitivity analysis 1 (per-protocol analysis) for the primary and secondary outcomes per study group during the PROMISS trial.

|  | **CON**  **N=91** | **PROT**  **N=36** | **PROT+TIMING**  **N=27** |
| --- | --- | --- | --- |
| **400-m walk test, s** |  |  |  |
| Baseline | 311.1 ± 9.3 | 394.1 ± 7.6 | 284.0 ± 9.0 |
| 6-month follow-up | 321.7 ± 13.0 | 291.9 ± 8.3 | 280.4 ± 7.2 |
| 6-month change, β (95% CI) | - | -10 (-21; 0.46) | -6 (-18; 6) |
| **SPPB summary score** |  |  |  |
| Baseline | 9.7 ± 0.17 | 10.1 ± 0.19 | 10.2 ± 0.19 |
| 6-month follow-up | 9.9 ± 0.19 | 10.1 ± 0.20 | 10.3 ± 0.24 |
| 6-month change, β (95% CI) | - | -0.23 (-0.67; 0.21) | -0.16 (-0.66; 0.34) |
| **Hand grip, kg** |  |  |  |
| Baseline | 29.2 ± 0.96 | 32.8 ± 1.77 | 28.4 ± 1.67 |
| 6-month follow-up | 28.3 ± 1.03 | 31.8 ± 1.72 | 27.8 ± 1.91 |
| 6-month change, β (95% CI) | - | 0.16 (-1.01; 1.33) | 0.18 (-1.14; 1.49) |
| **Leg extension strength, N** |  |  |  |
| Baseline | 321.3 ± 15.1 | 331.9 ± 27.8 | 309.4 ± 33.1 |
| 6-month follow-up | 302.9 ± 13.6 | 351.4 ± 24.7 | 298.1 ± 28.5 |
| 6-month change, β (95% CI) | - | 35.7 (7.8; 63.5) | 25.7 (-6.4; 57.9) |
| **Body fat percentage (BIA), %** |  |  |  |
| Baseline | 33.5 ± 0.74 | 31.5 ± 1.24 | 31.5 ± 1.95 |
| 6-month follow-up | 33.1 ± 0.83 | 30.4 ± 1.33 | 29.9 ± 1.96 |
| 6-month change, β (95% CI) | - | -0.15 (-1.77; 1.47) | -0.03 (-1.93; 1.86) |
| **Fat-free mass (BIA), kg** |  |  |  |
| Baseline | 51.3 ± 0.97 | 52.2 ± 1.77 | 51.3 ± 1.77 |
| 6-month follow-up | 51.8 ± 1.05 | 53.4 ± 2.15 | 52.7 ± 1.94 |
| 6-month change, β (95% CI) | - | 0.51 (-0.86; 1.88) | 0.49 (-1.11; 2.09) |
| **Fat percentage (BODPOD), %** |  |  |  |
| Baseline | 36.0 ± 1.18 | 36.2 ± 1.19 | 31.2 ± 3.2 |
| 6-month follow-up | 36.2 ± 1.30 | 37.1 ± 1.79 | 32.5 ± 2.98 |
| 6-month change, β (95% CI) | - | 0.39 (-1.15; 1.93) | 1.09 (-0.60; 2.79) |
| **Fat-free mass (BODPOD), kg** |  |  |  |
| Baseline | 50.5 ± 1.37 | 48.4 ± 2.25 | 50.8 ± 2.65 |
| 6-month follow-up | 50.9 ± 1.54 | 47.7 ± 2.32 | 51.7 ± 3.17 |
| 6-month change, β (95% CI) | - | -0.06 (-1.73; 1.60) | 0.87 (-0.95; 2.71) |
| **Self-reported mobility limitation** |  |  |  |
| Two consecutive reports at baseline and 3-month | 19 (20.9) | 7 (19.4) | 4 (14.8) |
| Two consecutive reports at 3-months 6-month | 15 (16.5) | 7 (19.4) | 4 (14.8) |
| 6-month change, β (95% CI) | - | 0.25 (-1.50; 1.99) | 0.55 (-1.20; 2.31) |

Data are mean ± standard error or N (%). Change scores are the 6-month follow-up value – the baseline value. Changes scores are presented as β (95% CI).

Abbreviations: β; regression coefficient adjusted for baseline measures of the outcomes, CI; confidence interval, BIA; bioelectrical impedance analysis, SPPB; Short Physical Performance Battery. Fat percentage (BODPOD) and Fat-free mass (BODPOD) were only measured in Dutch participants (N=79).

CON (reference category) = no intervention; PROT = personalized dietary advice aimed at increasing protein intake to at least 1.2 g/kg aBW/d; and PROT+TIMING = personalized dietary advice aimed at increasing protein intake to at least 1.2 g/kg aBW/d plus advice to time protein intake in close proximity of usual physical activity.

**Supplementary Table 5.** Sensitivity analysis 2 excluding the 80 participants with an extended month-6 clinic visit due to COVID-19 for the primary and secondary outcomes per study group during the PROMISS trial.

|  | **CON**  **N=65** | **PROT**  **N=64** | **PROT+TIMING**  **N=67** |
| --- | --- | --- | --- |
| **400-m walk test, s** |  |  |  |
| Baseline | 306.7 ± 10.0 | 311.5 ± 9.9 | 291.2 ± 5.2 |
| 6-month follow-up | 313.7 ± 12.0 | 300.8 ± 9.1 | 290.1 ± 4.8 |
| 6-month change, β (95% CI) | - | -17.7 (-30.5; -5.0) | -8.2 (-19.4; 3.0) |
| **SPPB summary score** |  |  |  |
| Baseline | 9.7 ± 0.18 | 9.7 ± 0.19 | 10.0 ± 0.14 |
| 6-month follow-up | 10.0 ± 0.20 | 9.9 ± 0.19 | 10.3 ± 0.16 |
| 6-month change, β (95% CI) | - | -0.12 (-0.49; 0.26) | -0.02 (-0.41; 0.36) |
| **Hand grip, kg** |  |  |  |
| Baseline | 29.3 ± 1.15 | 30.9 ± 1.23 | 29.8 ± 1.21 |
| 6-month follow-up | 28.4 ± 1.09 | 30.5 ± 1.27 | 29.0 ± 1.27 |
| 6-month change, β (95% CI) | - | 0.49 (-0.64; 1.63) | 0.16 (-0.95; 1.27) |
| **Leg extension strength, N** |  |  |  |
| Baseline | 309.9 ± 15.9 | 305.0 ± 17.8 | 292.7 ± 16.9 |
| 6-month follow-up | 298.4 ± 15.2 | 326.3 ± 17.4 | 304.7 ± 16.6 |
| 6-month change, β (95% CI) | - | 32.7 (2.0; 63.4) | 23.3 (-6.8; 53.4) |
| **Body fat percentage (BIA), %** |  |  |  |
| Baseline | 32.9 ± 0.82 | 32.1 ± 0.94 | 33.2 ± 0.90 |
| 6-month follow-up | 32.8 ± 0.89 | 32.7 ± 0.94 | 33.0 ± 0.94 |
| 6-month change, β (95% CI) | - | -0.33 (-1.67; 1.02) | -0.09 (-1.43; 1.25) |
| **Fat-free mass (BIA), kg** |  |  |  |
| Baseline | 51.7 ± 1.20 | 52.8 ± 1.26 | 52.0 ± 1.23 |
| 6-month follow-up | 51.9 ± 1.22 | 53.3 ± 1.37 | 52.4 ± 1.24 |
| 6-month change, β (95% CI) | - | 0.35 (-0.81; 1.50) | 0.27 (-0.89; 1.42) |
| **Fat percentage (BODPOD), %** |  |  |  |
| Baseline | 35.4 ± 1.36 | 35.6 ± 1.41 | 35.4 ± 1.70 |
| 6-month follow-up | 35.2 ± 1.48 | 35.2 ± 1.35 | 35.4 ± 1.62 |
| 6-month change, β (95% CI) | - | -0.19 (-2.27; 1.89) | 0.16 (-1.90; 2.21) |
| **Fat-free mass (BODPOD), kg** |  |  |  |
| Baseline | 50.3 ± 1.74 | 51.8 ± 1.70 | 49.9 ± 2.01 |
| 6-month follow-up | 51.2 ± 1.90 | 52.3 ± 1.85 | 51.4 ± 2.04 |
| 6-month change, β (95% CI) | - | -0.43 (-2.97; 2.10) | -0.59 (-1.84; 3.01) |
| **Self-reported mobility limitation** |  |  |  |
| Two consecutive reports at baseline and 3-month | 16 (24.0) | 15 (23.7) | 11 (16.7) |
| Two consecutive reports at 3-months 6-month | 12 (18.0) | 13 (20.1) | 13 (18.9) |
| 6-month change, β (95% CI) | - | 0.11 (-1.30; 1.53) | 0.50 (-0.84; 1.83) |

Data are mean ± standard error or N (%). Change scores are the 6-month follow-up value – the baseline value. Changes scores are presented as β (95% CI).

Abbreviations: β; regression coefficient adjusted for baseline measures of the outcomes, CI; confidence interval, BIA; bioelectrical impedance analysis, SPPB; Short Physical Performance Battery. Fat percentage (BODPOD) and Fat-free mass (BODPOD) were only measured in Dutch participants.

CON (reference category) = no intervention; PROT = personalized dietary advice aimed at increasing protein intake to at least 1.2 g/kg aBW/d; and PROT+TIMING = personalized dietary advice aimed at increasing protein intake to at least 1.2 g/kg aBW/d plus advice to time protein intake in close proximity of usual physical activity.

**Supplementary Table 6.** Sensitivity analysis 3 including only participants with complete data on the primary and secondary outcomes per study group during the PROMISS trial.

|  | **CON** | **PROT** | **PROT+TIMING** |
| --- | --- | --- | --- |
| **400-m walk test, s** | N=74 | N=80 | N=78 |
| Baseline | 313.9 ± 11.2 | 303.1 ± 5.8 | 288.2 ± 4.3 |
| 6-month follow-up | 321.7 ± 13.0 | 299.2 ± 6.0 | 290.1 ± 4.2 |
| 6-month change, β (95% CI) | - | -11.6 (-18.0; -4.3) | -5.8 (-13.2; 1.5) |
| **SPPB summary score** | N=74 | N=81 | N=79 |
| Baseline | 9.6 ± 0.19 | 9.8 ± 0.14 | 10.1 ± 0.13 |
| 6-month follow-up | 9.9 ± 0.19 | 10.0 ± 0.15 | 10.3 ± 0.13 |
| 6-month change, β (95% CI) | - | -0.10 (-0.45; 0.25) | -0.03 (-0.38; 0.32) |
| **Hand grip, kg** | N=74 | N=81 | N=79 |
| Baseline | 29.5 ± 1.09 | 31.0 ± 1.15 | 30.1 ± 1.12 |
| 6-month follow-up | 28.3 ± 1.02 | 30.2 ± 1.16 | 29.37 ± 1.16 |
| 6-month change, β (95% CI) | - | 0.39 (-0.54; 1.32) | 0.49 (-0.44; 1.44) |
| **Leg extension strength, N** | N=53 | N=61 | N=55 |
| Baseline | 321.5 ± 17.0 | 321.2 ± 17.8 | 334.9 ± 18.0 |
| 6-month follow-up | 313.1 ± 15.9 | 346.1 ± 18.2 | 345.5 ± 18.9 |
| 6-month change, β (95% CI) | - | 33.0 (11.4; 55.2) | 19.1 (-3.3; 41.5) |
| **Body fat percentage (BIA), %** | N=70 | N=78 | N=74 |
| Baseline | 33.6 ± 0.82 | 31.2 ± 0.81 | 32.5 ± 0.89 |
| 6-month follow-up | 33.1 ± 0.83 | 30.9 ± 0.81 | 32.2 ± 0.90 |
| 6-month change, β (95% CI) | - | 0.25 (-0.86; 1.35) | 0.24 (-0.88; 1.36) |
| **Fat-free mass (BIA), kg** | N=70 | N=78 | N=74 |
| Baseline | 51.5 ± 1.05 | 52.4 ± 1.21 | 53.1 ± 1.16 |
| 6-month follow-up | 51.8 ± 1.05 | 52.9 ± 1.29 | 53.6 ± 1.15 |
| 6-month change, β (95% CI) | - | 0.19 (-0.74; 1.12) | 0.14 (-0.81; 1.08) |
| **Fat percentage (BODPOD), %** | N=37 | N=39 | N=40 |
| Baseline | 36.0 ± 1.30 | 35.3 ± 1.25 | 34.6 ± 1.55 |
| 6-month follow-up | 36.2 ± 1.30 | 35.7 ± 1.10 | 34.8 ± 1.51 |
| 6-month change, β (95% CI) | - | 0.23 (-1.02; 1.47) | -0.04 (-1.28; 1.20) |
| **Fat-free mass (BODPOD), kg** |  |  |  |
| Baseline | 50.9 ± 1.44 | 50.2 ± 1.61 | 51.0 ± 1.80 |
| 6-month follow-up | 50.9 ± 1.53 | 50.2 ± 1.68 | 52.3 ± 1.94 |
| 6-month change, β (95% CI) | - | 0.10 (-1.40; 1.61) | 1.40 (-0.10; 2.90) |
| **Self-reported mobility limitation** | N=84 | N=89 | N=83 |
| Two consecutive reports at baseline and 3-month | 19 (22.6) | 16 (16.8) | 12 (14.5) |
| Two consecutive reports at 3-months 6-month | 15 (17.9) | 14 (15.7) | 14 (16.9) |
| 6-month change, β (95% CI) | - | 0.17 (-1.17; 1.52) | 0.44 (-0.86; 1.75) |

Data are mean ± standard error or N (%). Change scores are the 6-month follow-up value – the baseline value. Changes scores are presented as β (95% CI).

Abbreviations: β; regression coefficient adjusted for baseline measures of the outcomes, CI; confidence interval, BIA; bioelectrical impedance analysis, SPPB; Short Physical Performance Battery. Fat percentage (BODPOD) and Fat-free mass (BODPOD) were only measured in Dutch participants.

CON (reference category) = no intervention; PROT = personalized dietary advice aimed at increasing protein intake to at least 1.2 g/kg aBW/d; and PROT+TIMING = personalized dietary advice aimed at increasing protein intake to at least 1.2 g/kg aBW/d plus advice to time protein intake in close proximity of usual physical activity.

**Supplementary Table 7.** Sensitivity analysis 4; results of the cost-effectiveness analysis from the healthcare perspective.

| **Effect outcome^*^** | **Cost difference,**  **€ (95% CI)** | **Effect difference^§^**  ***-1 (95% CI)** | **ICER**  **€/ effect gained** | **Distribution of the cost-effectiveness plane** | | | |
| --- | --- | --- | --- | --- | --- | --- | --- |
|  |  |  |  | **North-East** | **South-East** | **South-West** | **North-West** |
| **Healthcare perspective** | | | | | | | |
| **Main analysis** | | | | | | | |
| PROT compared to CON | | | | | | | |
| Improvement in 400-m walk test, s | -117 (-790; 422) | 13 (2; 22) | -9 | 34% | 66% | 0% | 0% |
| QALY | -117 (-790; 422) | 0.006(-0.007; 0.020) | -18074 | 27% | 57% | 10% | 6% |
| PROT+TIMING compared to CON | | | | | | | |
| Improvement in 400-m walk test, s | -6 (-687; 783) | 7(-3; 13) | -1 | 43% | 45% | 7% | 5% |
| QALY | -6 (-687; 783) | 0.005 (-0.010; 0.017) | -1224 | 32% | 37% | 16% | 15% |
| **SA1 – Per protocol analysis** | | | | | | | |
| PROT compared to CON | | | | | | | |
| Improvement in 400-m walk test, s | -204 (-865; 409) | 9 (-21; 1) | -22 | 25% | 71% | 2% | 2% |
| QALY | -204 (-865; 409) | 0.009 (-0.005; 0.028) | -21524 | 22% | 68% | 5% | 5% |
| PROT+TIMING compared to CON | | | | | | | |
| 400-m walk test, s | -589 (-1346; -245) | 5 (-15; 6) | -116 | 0% | 78% | 22% | 0% |
| QALY | -589 (-1346; -245) | 0.006 (-0.014; 0.027) | -96072 | 0% | 76% | 24% | 0% |
| **SA2 – Excluding participants with an extended 6-month follow-up visit due to COVID-19.** | | | | | | | |
| PROT compared to CON | | | | | | | |
| Improvement in 400-m walk test, s | -296 (-1342; 160) | 20 (4; 31) | -15 | 18% | 82% | 0% | 0% |
| QALY | -296 (-1342; 160) | 0.010 (-0.006; 0.026) | -30217 | 15% | 74% | 8% | 3% |
| PROT+TIMING compared to CON | | | | | | | |
| Improvement in 400-m walk test, s | 20 (-879; 982) | 11 (-2; 18) | 2 | 49% | 47% | 2% | 2% |
| QALY | 20 (-879; 982) | 0.010 (-0.010; 0.025) | 2015 | 41% | 40% | 9% | 10% |
| **SA3 – Complete case analysis** | | | | | | | |
| PROT compared to CON | | | | | | | |
| Improvement in 400-m walk test, s | -45 (-407; 335) | 11 (3; 19) | -4 | 40% | 60% | 0% | 0% |
| QALY | -158 (-957; 438) | -0.007(-0.007; 0.021) | 23904 | 7% | 11% | 57% | 25% |
| PROT+TIMING compared to CON | | | | | | | |
| Improvement in 400-m walk test, s | 125 (-372; 1132) | 6 (-1; 13) | 20 | 59% | 36% | 2% | 3% |
| QALY | -34 (-782; 806) | -0.005 (-0.007; 0.019) | 6686 | 11% | 12% | 43% | 34% |

Data are mean (95% CI). **^§^**The effect outcome 400m walk test was multiplied by -1 to keep the cost-effectiveness plane interpretable. €, Euros CI, confidence interval; QALY, quality-adjusted life-years; ICER, incremental cost-effectiveness ratio.

CON (reference category) = no intervention; PROT = personalized dietary advice aimed at increasing protein intake to at least 1.2 g/kg aBW/d; and PROT+TIMING = personalized dietary advice aimed at increasing protein intake to at least 1.2 g/kg aBW/d plus advice to time protein intake in close proximity of usual physical activity.

SA1: sensitivity analysis 1, per-protocol analysis including participants from the two intervention groups who reached the protein target of at least 1.2 g/kg aBW/d at both 3- and 6-months follow-up vs. participants from CON. (total=154; CON N=91; PROT N=36, PROT+TIMING N=27).

SA2: sensitivity analysis 2 excluding participants with an extended month-6 clinic visit due to COVID-19 (total=196, CON N=65, PROT N=64, PROT+TIMING N=67).

SA3: sensitivity analysis 3 using complete cases for 400-m walk test and total healthcare costs (total=227, CON N=74, PROT N=77, PROT+TIMING N=76); and using complete cases for QALYs and total societal costs (total=253, CON N=84, PROT N=87, PROT+TIMING N=82).

**Supplementary Table 8.** Measured body weight and SNAQ appetite score and change values during the PROMISS randomized trial per study group.

|  | **CON**  **N=91** | **PROT**  **N=96** | **PROT+TIMING**  **N=89** |
| --- | --- | --- | --- |
| **Mean body weight, kg** |  |  |  |
| Baseline | 77.7 ± 1.13 | 76.5 ± 1.19 | 77.6 ± 1.17 |
| 3-month follow-up | 78.1 ± 1.10 | 76.8 ± 1.21 | 78.2 ± 1.18 |
| 3-month change, β (95% CI) | - | -0.06 (-0.57; 0.44) | 0.12 (-0.40; 0.66) |
| 6-month follow-up | 77.9 ± 1.13 | 76.9 ± 1.23 | 78.0 ± 1.19 |
| 6-month change, β (95% CI) | - | 0.14 (-0.67; 0.96) | 0.19 (-0.61; 0.98) |
| **Appetite score (max 20)** |  |  |  |
| Baseline | 17.0 ± 0.15 | 17.1 ± 0.14 | 17.2 ± 0.14 |
| 6-month follow-up | 17.1 ± 0.16 | 17.1 ± 0.16 | 17.2 ± 0.15 |
| 6-month change, β (95% CI) | - | -0.09 (-0.49; 0.31) | -0.17 (-0.57; 0.24) |

Data are mean ± standard error Change scores are the 3- and 6-month follow-up value – the baseline value.

Change scores are presented as β (95% CI). β; regression coefficient. CI; confidence interval.

CON (reference category) = no intervention; PROT = personalized dietary advice aimed at increasing protein intake to at least 1.2 g/kg aBW/d; and PROT+TIMING = personalized dietary advice aimed at increasing protein intake to at least 1.2 g/kg aBW/d plus advice to time protein intake in close proximity of usual physical activity.

**Supplementary Table 9.** Physical activity assessed by accelerometry at 3- and 6-month follow-up per study group.

|  | **CON**  **N=91** | **PROT**  **N=96** | **PROT+TIMING**  **N=89** |
| --- | --- | --- | --- |
| **Count per minute over 24-h time-window** |  |  |  |
| Baseline | 252 ± 11 | 276 ± 14 | 285 ± 14 |
| 3-month follow-up | 235 ± 11 | 247 ± 11 | 272 ± 14 |
| 3-month change, β (95% CI) | - | -14 (-32; 5) | 6 (-13; 25) |
| 6-month follow-up | 233 ± 12 | 249 ± 12 | 254 ± 14 |
| 6-month change, β (95% CI) | - | -11 (-36; 14) | -3 (-28; 22) |
| **Total counts per day** |  |  |  |
| Baseline | 2175095 ± 98098 | 2385239 ± 117098 | 2465488 ± 117048 |
| 3-month follow-up | 2025479 ± 95165 | 2128859 ± 98143 | 2347979 ± 119647 |
| 3-month change, β (95% CI) | - | -118327 (-279673; 43019) | 49135 (-115741; 214012) |
| 6-month follow-up | 2010802 ± 106725 | 2151191 ± 104217 | 2190840 ± 117320 |
| 6-month change, β (95% CI) | - | -94062 (-313052; 124926) | -27151 (-247095; 192792) |
| **Counts per minute including only physical activity measured over 24-hour time-window^a^** |  |  |  |
| Baseline | 1533 ± 50 | 1562 ± 48 | 1628 ± 53 |
| 3-month follow-up | 1427 ± 47 | 1473 ± 47 | 1572 ± 56 |
| 3-month change, β (95% CI) | - | -19 (-103; 65) | 35 (--52; 121) |
| 6-month follow-up | 1463 ± 57 | 1475 ± 46 | 1541 ± 56 |
| 6-month change, β (95% CI) | - | -61 (-166; 44) | -4 (-110; 102) |
| **Total steps per day** |  |  |  |
| Baseline | 5264 ± 291 | 5503 ± 284 | 5863 ± 257 |
| 3-month follow-up | 4871 ± 269 | 5092 ± 254 | 5728 ± 246 |
| 3-month change, β (95% CI) | - | -46 (-500; 407) | 232 (-233; 697) |
| 6-month follow-up | 4873 ± 268 | 5155 ± 269 | 5273 ± 225 |
| 6-month change, β (95% CI) | - | 14 (-539; 566) | -73 (-632; 485) |
| **Time in non-sedentary activities, min^b^** |  |  |  |
| Baseline | 100 ± 3.8 | 107 ± 4.6 | 108 ± 3.8 |
| 3-month follow-up | 94 ± 3.8 | 95 ± 3.8 | 105 ± 3.8 |
| 3-month change, β (95% CI) | - | -5 (-12; 1) | 3 (-4; 10) |
| 6-month follow-up | 95 ± 5.2 | 99 ± 4.1 | 100 ± 3.7 |
| 6-month change, β (95% CI) | - | -3 (-13; 7) | -0.1 (-10; 10) |

^a^This includes any counts above 100 counts per minute

^b^Includes walking, running, stepping, biking and move.

Data are mean ± standard error Change scores are the 3- and 6-month follow-up value – the baseline value.

Change scores are presented as β (95% CI). β; regression coefficient. CI; confidence interval.

CON (reference category) = no intervention; PROT = personalized dietary advice aimed at increasing protein intake to at least 1.2 g/kg aBW/d; and PROT+TIMING = personalized dietary advice aimed at increasing protein intake to at least 1.2 g/kg aBW/d plus advice to time protein intake in close proximity of usual physical activity.

**Supplemental Figure 1. Cost-effectiveness planes (CE-plane) from the healthcare perspective.**


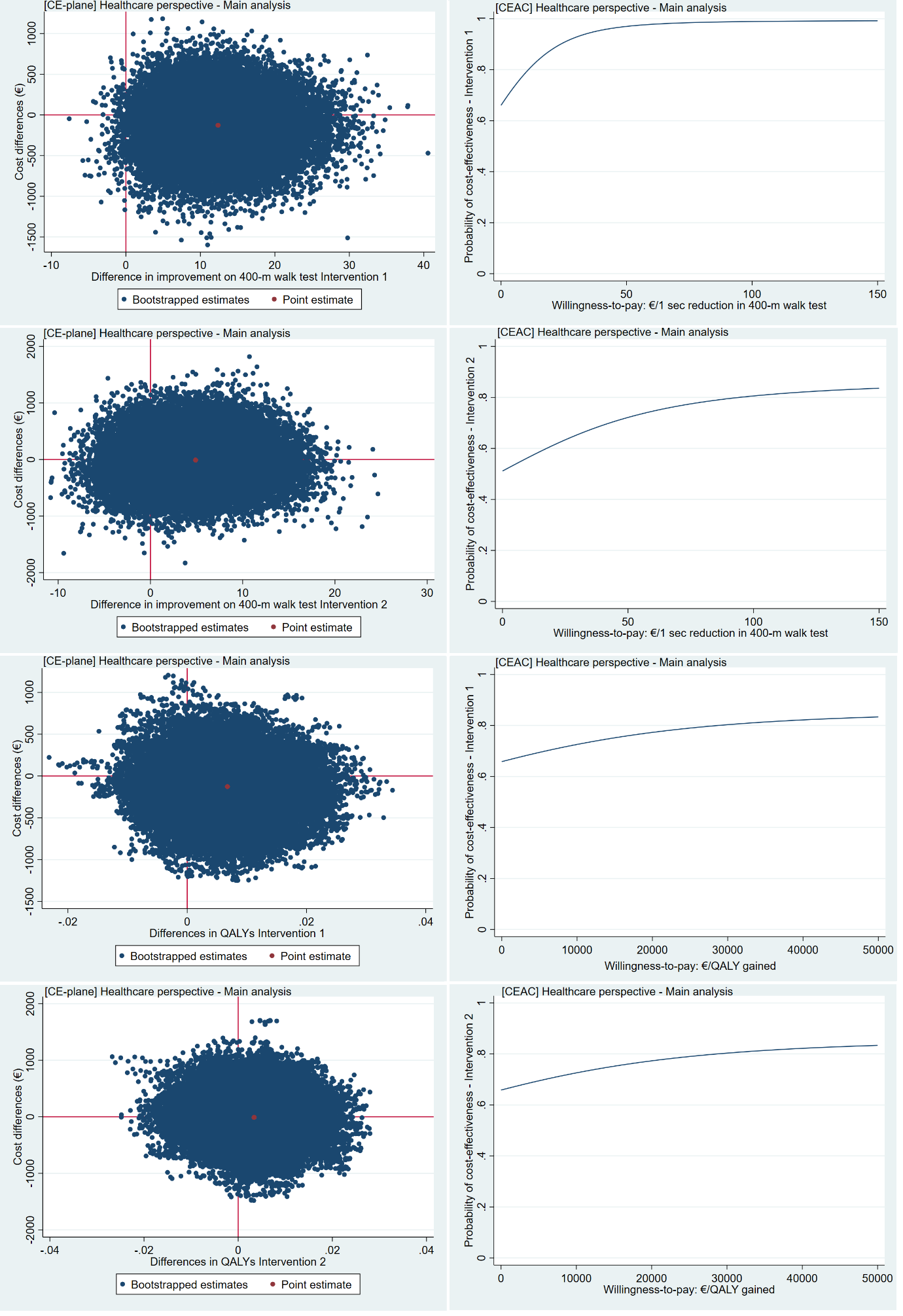


Cost-effectiveness planes (CE-plane) from the healthcare perspective showing the incremental cost-effectiveness ratio point estimate (red dot) and the distribution of the 5,000 replications of the bootstrapped cost-effective pairs (blue dots). Cost-effectiveness acceptability curves (CEAC) indicating the probability of cost-effectiveness (y-axis) for different willingness-to-pay (WTP) thresholds per unit of effect gained (x-axis).

**PROMISS trial group**

**PROMISS project coordination, Vrije Universiteit Amsterdam, Department of Health Sciences, the Netherlands**

Prof. Marjolein Visser, PhD – Principle investigator of the PROMISS project

Prof. Ingeborg Brouwer, PhD – Project manager of the PROMISS project

Margreet Olthof, PhD – Financial manager of the PROMISS project

Rachel Vijlbrief, MSc – Assistant project manager of the PROMISS project

*Trial sites*

Vrije Universiteit Amsterdam, the Netherlands

Hanneke Wijnhoven, PhD - local principal investigator

Ilse Reinders, PhD – local co-principal investigator

Nanouk Bakker Schut, BSc – research intern

Judith Bosmans, PhD – researcher

Mariska Bout, BSc – dietician and research assistant

Ingeborg Brouwer, PhD – researcher

Nona Kerremans – research intern

Lothar Kuijper, PhD – researcher

Margreet Olthof, PhD – researcher

Marjon Veeke, MSC – dietician and research assistant

Rachel Vijlbrief, MSc – researcher

Marjolein Visser, PhD – researcher

Merel Vrijmoeth, MSc – dietician and research assistant

Laura Winkens, MSc – researcher

University of Helsinki

Merja Suominen, PhD – local principal investigator

Satu Jyväkorpi, PhD – local co-principal investigator

Kirsi Ali-Kovero, BSc – research assistant

Johannes Anttila, BSC – research intern

Aliisa Hyvönen, MSc – dietician and research assistant

Henriikka Jussila, BSc – research intern

Anna-Maria Piipponen, BSc – research intern and research assistant

Riikka Niskanen, MSc – dietician and research assistant

Kaisu Pitkälä, PhD – researcher

Heli Salmenius-Suominen – researcher

*Ancillary studies*

Persuasive technology study

Michel Klein, PhD – principal investigator of the persuasive technology study, *Vrije Universiteit Amsterdam, the Netherlands*

Michèle Go, BSc – research intern, *Vrije Universiteit Amsterdam, the Netherlands*

Laura van der Lubbe, MSc – researcher, *Vrije Universiteit Amsterdam, the Netherlands*

Linh Tran, BSc – research intern, *Vrije Universiteit Amsterdam, the Netherlands*

Microbiota study and fMRI study

Max Nieuwdorp, MD, PhD – principal investigator of the microbiota study and fMRI study, *Amsterdam UMC, location AMC and location VUmc, Amsterdam, the Netherlands*

Fredrik Bäckhed, MD, PhD – researcher, *University of Gothenburg, Gothenburg, Sweden and University of Copenhagen, Copenhagen, Denmark*

Kristien Fluitman, MD – researcher, *Amsterdam UMC, location VUmc, Amsterdam, the Netherlands*

Bart Keijser, PhD – researcher, *TNO earth, Zeist, the Netherlands and Academic Center for Dentistry Amsterdam, the Netherlands*

Charlotte van Ruiten, MD – substitute K. Fluitman, *Amsterdam UMC, location VUmc, Amsterdam, the Netherlands*

Richard IJzerman, MD, PhD – researcher, *Amsterdam UMC, location VUmc, Amsterdam, the Netherlands*

References
