# Supplementary material for: The cost effectiveness of personalized dietary advice to increase protein intake in older adults with lower habitual protein intake: a randomized controlled trial
Source: Eur J Nutr. 2021 Oct 5;61(1):505–20. doi: 10.1007/s00394-021-02675-0 (PMC8490609; doi:10.1007/s00394-021-02675-0)
Supplement: Supplementary file 2 — Supplementary file2 (PDF 174 KB) [file 394_2021_2675_MOESM2_ESM.pdf]

**The cost-effectiveness of personalized dietary advice to increase protein intake on change in physical functioning in older adults with lower habitual protein intake**

A Randomized Controlled Trial

**European Journal of Nutrition**

Ilse Reinders[1], Marjolein Visser[1], Satu K. Jyväkorpi[2], Riikka T. Niskanen[2], Judith E. Bosmans[1], Ângela Jornada Ben[1], Ingeborg A. Brouwer[1], Lothar D. Kuijper[1], Margreet R. Olthof[1], Kaisu H. Pitkälä[2], Rachel Vijlbrief[1], Merja H. Suominen[2] and Hanneke A.H. Wijnhoven[1].

[1]Department of Health Sciences, Faculty of Science, and the Amsterdam Public Health research institute, Vrije Universiteit Amsterdam, The Netherlands; [2] University of Helsinki, Department of General Practice and Primary Health Care, and Helsinki University Central Hospital, Unit of Primary Health Care, Finland.

**Corresponding author:**

Hanneke Wijnhoven

E-mail: hanneke.wijnhoven@vu.nl

NOT TO BE REPRODUCED

Under each heading, please tick the ONE box that best describes your health TODAY

**MOBILITY**
☐I have no mobility problems
☐I have slight mobility problems
☐I have moderate mobility problems
☐I have severe mobility problems
☐I am unable to move.

**SELF-CARE**
☐I have no problems washing or dressing myself
☐I have slight problems washing or dressing myself
☐I have moderate problems washing or dressing myself
☐I have severe problems washing or dressing myself
☐I am unable to wash or dress myself

**USUAL ACTIVITIES** *(e.g. work, study, housework, family or leisure activities)*
☐I have no problems doing my usual activities☐
☐I have slight problems doing my usual activities☐
☐I have moderate problems doing my usual activities
☐I have severe problems doing my usual activities☐
☐I am unable to do my usual activities

**PAIN / DISCOMFORT**
☐I have no pain or discomfort☐
☐I have slight pain or discomfort
☐I have moderate pain or discomfort
☐I have severe pain or discomfort☐
☐I have extreme pain or discomfort

**ANXIETY / DEPRESSION**
☐I am not anxious or depressed
☐I am slightly anxious or depressed☐
☐I am moderately anxious or depressed
☐I am severely anxious or depressed
☐I am extremely anxious or depressed

NOT TO BE REPRODUCED

Script: ***We would like to know how good or bad your health is TODAY.***
***This scale is numbered from 0 to 100. 100 means the best health you can imagine. 0 means the worst health you can imagine. Mark an X on the scale to indicate how your health is TODAY.***

***Now, please write the number you marked on the scale in the box below.***

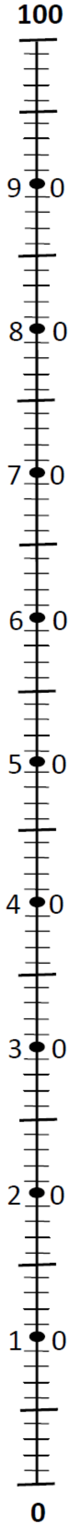


**YOUR HEALTH TODAY:** ☐

NOT TO BE REPRODUCED
